# Living risk prediction algorithm (QCOVID) for risk of hospital admission and mortality from coronavirus 19 in adults: national derivation and validation cohort study

**DOI:** 10.1136/bmj.m3731

**Published:** 2020-10-21

**Authors:** Ash K Clift, Carol A C Coupland, Ruth H Keogh, Karla Diaz-Ordaz, Elizabeth Williamson, Ewen M Harrison, Andrew Hayward, Harry Hemingway, Peter Horby, Nisha Mehta, Jonathan Benger, Kamlesh Khunti, David Spiegelhalter, Aziz Sheikh, Jonathan Valabhji, Ronan A Lyons, John Robson, Malcolm G Semple, Frank Kee, Peter Johnson, Susan Jebb, Tony Williams, Julia Hippisley-Cox

**Affiliations:** 1 Nuffield Department of Primary Care Health Sciences, Radcliffe Observatory Quarter, Oxford OX2 6GG, UK; 2 Division of Primary Care, School of Medicine, University of Nottingham, Nottingham, UK; 3 Faculty of Epidemiology and Population Health, London School of Hygiene and Tropical Medicine, London, UK; 4 Usher Institute, University of Edinburgh, Edinburgh, UK; 5 UCL Institute of Epidemiology and Health Care, University College London, London, UK; 6 UCL Institute for Health Informatics, University College London, London, UK; 7 Centre for Tropical Medicine and Global Health, University of Oxford, Oxford, UK; 8 Office of the Chief Medical Officer, Department of Health and Social Care, London, UK; 9 NHS Digital, Leeds, UK; 10 Diabetes Research Centre, University of Leicester, Leicester, UK; 11 Winton Centre for Risk and Evidence Communication, Faculty of Mathematics, University of Cambridge, Cambridge, UK; 12 NHS England, London, UK; 13 Swansea University, Swansea, UK; 14 Centre for Primary Care and Public Health, Queen Mary University of London, London, UK; 15 Institute of Infection, Veterinary and Ecological Sciences, University of Liverpool, Liverpool, UK; 16 Centre for Public Health, Queen’s University Belfast, Belfast, UK; 17 Association of Local Authority Medical Advisors, London, UK; 18 Imperial College London, London, UK

## Abstract

**Objective:**

To derive and validate a risk prediction algorithm to estimate hospital admission and mortality outcomes from coronavirus disease 2019 (covid-19) in adults.

**Design:**

Population based cohort study.

**Setting and participants:**

QResearch database, comprising 1205 general practices in England with linkage to covid-19 test results, Hospital Episode Statistics, and death registry data. 6.08 million adults aged 19-100 years were included in the derivation dataset and 2.17 million in the validation dataset. The derivation and first validation cohort period was 24 January 2020 to 30 April 2020. The second temporal validation cohort covered the period 1 May 2020 to 30 June 2020.

**Main outcome measures:**

The primary outcome was time to death from covid-19, defined as death due to confirmed or suspected covid-19 as per the death certification or death occurring in a person with confirmed severe acute respiratory syndrome coronavirus 2 (SARS-CoV-2) infection in the period 24 January to 30 April 2020. The secondary outcome was time to hospital admission with confirmed SARS-CoV-2 infection. Models were fitted in the derivation cohort to derive risk equations using a range of predictor variables. Performance, including measures of discrimination and calibration, was evaluated in each validation time period.

**Results:**

4384 deaths from covid-19 occurred in the derivation cohort during follow-up and 1722 in the first validation cohort period and 621 in the second validation cohort period. The final risk algorithms included age, ethnicity, deprivation, body mass index, and a range of comorbidities. The algorithm had good calibration in the first validation cohort. For deaths from covid-19 in men, it explained 73.1% (95% confidence interval 71.9% to 74.3%) of the variation in time to death (R^2^); the D statistic was 3.37 (95% confidence interval 3.27 to 3.47), and Harrell’s C was 0.928 (0.919 to 0.938). Similar results were obtained for women, for both outcomes, and in both time periods. In the top 5% of patients with the highest predicted risks of death, the sensitivity for identifying deaths within 97 days was 75.7%. People in the top 20% of predicted risk of death accounted for 94% of all deaths from covid-19.

**Conclusion:**

The QCOVID population based risk algorithm performed well, showing very high levels of discrimination for deaths and hospital admissions due to covid-19. The absolute risks presented, however, will change over time in line with the prevailing SARS-C0V-2 infection rate and the extent of social distancing measures in place, so they should be interpreted with caution. The model can be recalibrated for different time periods, however, and has the potential to be dynamically updated as the pandemic evolves.

## Introduction

The first cases of severe acute respiratory syndrome coronavirus 2 (SARS-CoV-2) infection were reported in the UK on 24 January 2020, with the first death from coronavirus disease 2019 (covid-19) on 28 February 2020. As of 18 August 2020, more than 41 000 deaths from covid-19 had occurred in the UK and more than 773 000 deaths globally.^1^ In the initial absence of any vaccination or prophylactic or curative treatments, the UK government implemented social distancing and shielding measures to suppress the rate of infection and protect vulnerable people, thereby trying to minimise the risk of serious adverse outcomes.^2^ ^3^

Emerging evidence throughout the course of the pandemic, initially from case series and then from cohorts of patients with confirmed SARS-CoV-2 infection, has shown associations of age, sex, certain comorbidities, ethnicity, and obesity with adverse covid-19 outcomes such as hospital admission or death.^4^ ^5^ ^6^ ^7^ ^8^ ^9^ ^10^ ^11^ The knowledge base regarding risk factors for severe covid-19 is growing. As many countries are cautiously attempting to ease “lockdown” measures or reintroduce measures if rates are rising, an opportunity exists to develop more nuanced guidance based on predictive algorithms to inform risk management decisions.^12^ Better knowledge of individuals’ risks could also help to guide decisions on mitigating occupational exposure and in targeting of vaccines to those most at risk. Although some prediction models have been developed, a recent systematic review found that they all have a high risk of bias and that their reported performance is optimistic.^13^

The use of primary care datasets with linkage to registries such as death records, hospital admissions data, and covid-19 testing results represents a novel approach to clinical risk prediction modelling for covid-19. It provides accurately coded, individual level data for very large numbers of people representative of the national population. This approach draws on the rich phenotyping of individuals with demographic, medical, and pharmacological predictors to allow robust statistical modelling and evaluation. Such linked datasets have an established track record for the development and evaluation of established clinical risk models, including those for cardiovascular disease, diabetes, and mortality.^14^ ^15^ ^16^ We aimed to develop and validate population based prediction models to estimate the risks of becoming infected with and subsequently dying from covid-19 and of becoming infected and subsequently admitted to hospital with covid-19. The model we have developed is designed to be applied across the adult population so that it can be used to enable risk stratification for public health purposes in the event of a “second wave” of the pandemic, to support shared management of risk and occupational exposure, and in early targeting of vaccines to people most at risk. An ongoing companion study will externally validate the models, using datasets across all four nations of the UK, and will be reported separately.

## Methods

This study was commissioned by the Chief Medical Officer for England on behalf of the UK Government, who asked the New and Emerging Respiratory Virus Threats Advisory Group (NERVTAG) to establish whether a clinical risk prediction model for covid-19 could be developed in line with the emerging evidence. The protocol has been published.^17^ The study was conducted in adherence with TRIPOD^18^ and RECORD^19^ guidelines and with input from our patient advisory group.

### Study design and data sources

We did a cohort study of primary care patients using the QResearch database (version 44). QResearch was established in 2002 and has been extensively used for the development of risk prediction algorithms across the National Health Service (NHS) and for epidemiological research. By April 2020, 1205 practices in England were contributing to QResearch, covering a population of 10.5 million patients. The database is linked at individual patient level, using a project specific pseudonymised NHS number, to hospital admissions data (including intensive care unit data), positive results from covid-19 real time reverse transcriptase polymerase chain reaction tests held by Public Health England, cancer registrations (including detailed radiotherapy and systemic chemotherapy records), the national covid-19 shielded patient list in England, and mortality records held by NHS Digital.

We identified a cohort of people aged 19-100 years registered with participating general practices in England on 24 January 2020. We excluded patients (approximately 0.1%) who did not have a valid NHS number. Patients entered the cohort on 24 January 2020 (date of first confirmed case of covid-19 in the UK) and were followed up until they had the outcome of interest or the end of the first study period (30 April 2020), which was the date up to which linked data were available at the time of the derivation of the model, or the second time period (1 May 2020 until 30 June 2020) for the temporal cohort validation.

### Outcomes

The primary outcome was time to death from covid-19 (either in hospital or outside hospital), defined as confirmed or suspected death from covid-19 as per the death certification or death occurring in an individual with confirmed SARS-CoV-2 infection at any time in the period 24 January to 30 April 2020. The secondary outcome was time to hospital admission with covid-19, defined as an ICD-10 (International Classification of Diseases, 10th revision) code for either confirmed or suspected covid-19 or new hospital admission associated with a confirmed SARS-CoV-2 infection in the study period.

### Predictor variables

We selected candidate predictor variables on the basis of the presence of existing clinical vulnerability group criteria (table 1), associations with outcomes in other respiratory diseases, or hypothesised to be linked to adverse outcomes on clinical/biological plausibility and likely to be available for implementation. They are summarised in box 1 and supplementary box A. We defined variables according to information recorded using Read Codes in general practices’ electronic health records at the start of the study period. The exception to this was information on chemotherapy, radiotherapy, and transplants, which was based on linked hospital records.

**Table 1:** Original population level risk stratification method as exercised in UK*

Clinical risk level	Advice	Criteria	Identification and inclusion
Clinically extremely vulnerable (high risk)	Shielding (stay at home and stringently avoid any personal/face-to-face contact)	High risk conditions as established by clinical expert group decisions based on available evidence at time. Dynamic group of approximately 2.2 million people in England	Method 1: core group of patients identified by NHS Digital and contacted centrally by NHS England
			Method 2: additional patients in particular medical sub-specialties not identifiable centrally
			Method 3: additional patients identified by secondary care specialists using clinical judgment
			Method 4: additional patients identified in primary care using clinical judgment
Clinically vulnerable (medium risk)	Follow stringent social distancing measures	Vulnerable group of approximately 16 million people in England, based on eligibility for influenza vaccination due to age ≥70, pregnancy, or comorbidity	NA
Remainder of population (low risk)	Follow mandatory social distancing measures and “lockdown” measures, but no specific clinical advice	NA	NA

NA=not applicable.

* Shielding and stringent social distancing are both interventions designed to reduce risk of exposure to SARS-CoV-2, but classification of risk relates to risk of complicated or fatal disease if infected and not risk of becoming infected.

Box 1 Candidate predictor variables examined during model development*

**Demographic**

- Age in years (continuous)
- Townsend deprivation score (continuous)—This is an area level continuous score based on the patient’s postcode.^20^ Originally developed by Townsend,^20^ it includes unemployment (as a percentage of those aged ≥16 who are economically active), non-car ownership (as a percentage of all households), non-home ownership (as a percentage of all households), and household overcrowding. These variables are measured for a given area of approximately 120 households, via the 2011 census, and combined to give a Townsend score for that area. A greater Townsend score implies a greater level of deprivation
- Ethnicity in nine categories (White, Indian, Pakistani, Bangladeshi, Other Asian, Caribbean, Black African, Chinese, other ethnic group)
- Domicile in three categories: homeless, care home residence (nursing or residential), other

**Lifestyle**

- Smoking status in five categories (non-smoker, ex-smoker, 1-10 per day, 11-19 per day, ≥20 per day)
- Body mass index in kg/m^2^ (continuous)
- Crack cocaine and injected drug use

**Conditions on current shielding patient list**

- Solid organ transplant recipient on long term immune suppression treatment
- Cancers:
  - Active chemotherapy
  - Radical radiotherapy for lung cancer
  - Blood/bone marrow cancer at any treatment stage
  - Immunotherapy or continuing antibody treatment
  - Targeted cancer treatments that affect immune system (PARP inhibitor or PKI)
  - Bone marrow or stem cell transplant in previous 6 months or remain on immunosuppression
- Immunosuppression sufficiently increasing infection risk
- Severe respiratory disease:
  - Severe asthma (≥3 prescribed courses of steroids in preceding 12 months)
  - Severe COPD (≥3 prescribed courses of steroids in preceding 12 months)
  - Cystic fibrosis, interstitial lung disease, sarcoidosis, non-cystic fibrosis bronchiectasis, or pulmonary hypertension
- Rare diseases or inborn errors of metabolism:
  - Severe combined immunodeficiency
  - Homozygous sickle cell disease
- Pregnant with significant heart disease:
  - Acquired or congenital

**Conditions moderately associated with increased risk of complications as per current NHS guidance**

- Chronic, non-severe respiratory disease:
  - Asthma
  - COPD (emphysema and chronic bronchitis)
  - Extrinsic allergic alveolitis
- Chronic kidney disease (CKD):
  - CKD stage 3 or 4
  - End stage renal failure requiring dialysis
  - End stage renal failure ever undergoing a transplant
- Cardiac disease:
  - Congestive cardiac failure
  - Valvular heart disease
- Chronic liver disease:
  - Chronic infective hepatitis (hepatitis B or C)
  - Alcohol related liver disease
  - Primary biliary cirrhosis
  - Primary sclerosing cholangitis
  - Haemochromatosis
- Chronic neurological conditions:
  - Epilepsy
  - Parkinson’s disease
  - Motor neurone disease
  - Cerebral palsy
  - Dementia (Alzheimer’s, vascular, frontotemporal)
  - Down’s syndrome
- Diabetes mellitus:
  - Type 1
  - Type 2
- Conditions or treatments that predispose to infection (eg, steroid treatment):
  - Rheumatoid arthritis
  - Systemic lupus erythematosus
  - Ankylosing spondylitis or other inflammatory arthropathy (eg, psoriatic arthritis)
  - Connective tissue disease (eg, Ehlers-Danlos syndrome, scleroderma, Sjögren’s syndrome)
  - Polymyositis or dermatomyositis
  - Vasculitis (eg, giant cell arteritis, polyarteritis nodosa, Behçet’s syndrome)

**Other medical conditions that investigators hypothesised to confer elevated risk**

- Cardiovascular disease:
  - Atrial fibrillation
  - Cardiovascular events (myocardial infarction, stroke, angina, transient ischaemic attack)
  - Peripheral vascular disease
  - Treated hypertension
- Hyperthyroidism
- Chronic pancreatitis
- Cirrhosis (if not above; eg, non-alcoholic fatty liver disease)
- Malabsorption:
  - Coeliac disease
  - Steatorrhoea
  - Blind loop syndrome
- Peptic ulcer (gastric or duodenal)
- Learning disability
- Osteoporosis
- Fragility fracture (hip, spine, shoulder, or wrist fracture)
- Severe mental illness:
  - Bipolar affective disorder
  - Psychosis
  - Schizophrenia or schizoaffective disorder
  - Severe depression
- HIV infection
- Hyposplenism
- Sickle cell disease
- Sphingolipidoses (eg, Tay-Sachs disease)
- History of venous thromboembolism
- Tuberculosis

**Concurrent medications**

- Drugs affecting the immune response, including systemic chemotherapy based on hospital data
- Drugs affecting the immune system prescribed in primary care (focus on *BNF* chapter 8.2)
- Long acting β agonists
- Long acting muscarinic antagonists
- Inhaled corticosteroids

COPD=chronic obstructive pulmonary disease; PARP=poly ADP ribose polymerase; PKI=protein kinase A inhibitor.*Based on data recorded in general practice record linked to hospital information on chemotherapy, radiotherapy, and transplants

### QCOVID model development

We randomly allocated 75% of practices to the derivation dataset, which we used to develop the models. We evaluated the models’ performance in the remaining 25% of practices (the validation set). All models were fitted separately in men and women. The outcomes of interest are subject to competing risks. For the primary outcome of death from covid-19, the competing risk is death due to other causes. For the secondary outcome of hospital admission, the competing risk is death from any cause before admission. We fitted a sub-distribution hazard (Fine and Gray^21^) model for each outcome to account for competing risks. Individuals who did not have the outcome of interest were censored at the study end date, including those who had a competing event.

For all predictor variables, we used the most recently available value at the entry date (24 January 2020). We used second degree fractional polynomials to model non-linear relations for continuous variables (age, body mass index, and Townsend material deprivation score, an area level score based on postcode^20^). Initially, we fitted a complete case analysis by using a model within the derivation data to derive the fractional polynomial terms. For indicators of comorbidities and medication use, we assumed the absence of recorded information to mean absence of the factor in question. Data were missing in four variables: ethnicity, Townsend score, body mass index, and smoking status. We used multiple imputation with chained equations under the missing at random assumption to replace missing values for these variables. For computational efficiency, we used a combined imputation model for both outcomes. The imputation model was fitted in the derivation data and included predictor variables, the Nelson-Aalen estimators of the baseline cumulative sub-distribution hazard, and the outcome indicators (death from covid-19 and hospital admission with covid-19). We carried out five imputations. Each analysis model was fitted in each of the five imputed datasets. We used Rubin’s rules to combine the model parameter estimates and the baseline cumulative incidence estimates across the imputed datasets.

We initially sought to fit models using all predictor variables. Owing to sparse cells, some conditions were combined if clinically similar in nature (such as rare neurological disorders). We examined interactions between body mass index and ethnicity and interactions between predictor variables and age, focusing on predictor variables that apply across the age range (asthma, epilepsy, diabetes, severe mental illness). We explored the use of penalised models (LASSO) to screen variables for inclusion, but this retained all the predictor variables and most interaction terms.^17^ In line with the protocol, we subsequently removed a small number of variables with low numbers of events and adjusted (sub-distribution) hazard ratios close to 1 (as these will have minimal effect on predicted risks) or with uncertain clinical credibility, defined as counterintuitive results in light of the emerging literature. Lastly, we combined regression coefficients from the final models with estimates of the baseline cumulative incidence function evaluated at 97 days to derive risk equations for each outcome. We used all the available data in the database.

### Model evaluation

We did all model evaluation using the validation data with two separate periods of follow-up. The first validation study period was the same as for the derivation cohort: 24 January to 30 April 2020. The second temporal validation covered the subsequent period of 1 May 2020 to 30 June 2020. This was carried out with the same validation cohort except for exclusion of patients who died during 24 January to 30 April 2020. In the validation cohort, we fitted an imputation model to replace missing values for ethnicity, body mass index, Townsend score, and smoking status. This excluded the outcome indicators and Nelson-Aalen terms, as the aim was to use covariate data to obtain a prediction as if the outcome had not been observed to reflect intended use.

We applied the final risk equations developed from the derivation dataset to men and women in the validation dataset and evaluated R^2^ values, Brier scores, and measures of discrimination and calibration for the two time periods.^22^ ^23^ ^24^ R^2^ values refer to the proportion of variation in survival time explained by the model. Brier scores measure predictive accuracy, where lower values indicate better accuracy.^25^ D statistics (a discrimination measure that quantifies the separation in survival between patients with different levels of predicted risks) and Harrell’s C statistics (a discrimination metric that quantifies the extent to which people with higher risk scores have earlier events) were evaluated at 97 days (the maximum follow-up period available at the time of the derivation of the model) and 60 days for the second temporal validation, with corresponding 95% confidence intervals.^26^ We assessed model calibration by comparing mean predicted risks with observed risks by twentieths of predicted risk for each of the validation cohorts. Observed risks were derived in each of the 20 groups by using non-parametric estimates of the cumulative incidences. Additionally, we did a recalibration for the mortality outcome, using the method proposed by Booth et al by updating the baseline survivor function based on the temporal validation cohort with the prognostic index as an offset term.^27^ We also applied the algorithms to the validation cohort for the first time period to define the centile thresholds based on absolute risk. We also defined centiles of relative risk (defined as the ratio of the individual’s predicted absolute risk to the predicted absolute risk for a person of the same age and sex with a white ethnicity, body mass index of 25, and mean deprivation score with no other risk factors).

We calculated the performance metrics in the whole validation cohort and in the following pre-specified subgroups: within age groups (19-39, 40-49, 50-59, 60-69, 70-79, ≥80 years), within nine ethnic groups, and within each of the 10 English regions (where numbers allowed). In response to open peer review of the published protocol,^17^ we evaluated performance by calculating Harrell’s C statistics in individual general practices and combining the results using a random effects meta-analysis.^28^

### Patient and public involvement

Patients were involved in setting the research question and in developing plans for design and implementation of the study. Patients were asked to aid in interpreting and disseminating the results.

## Results

### Overall study population

Overall, 1205 practices in England met our inclusion criteria. Of these, 910 practices were randomly assigned to the derivation dataset and 295 to the validation cohort. The practices had 8 256 158 registered patients aged 19-100 years on 24 January 2020. We included 6 083 102 of these in the derivation cohort, and the validation dataset comprised 2 173 056 people.

### Baseline characteristics

Table 2 shows the baseline characteristics of patients in the derivation cohort. Of these patients, 3 035 409 (49.9%) were men and 990 799 (16.3%) were of black, Asian, or other minority ethnic (BAME) background.

**Table 2:** Demographic and medical characteristics of derivation cohort and cohort members with outcomes. Values are numbers (percentages) unless stated otherwise

Characteristic	Derivation cohort—total (n=6 083 102)	Derivation cohort—covid-19 deaths (n=4384)	Derivation cohort—covid-19 admission (n=10 776)
Male sex	3 035 409 (49.90)	2517 (57.41)	5962 (55.33)
Mean (SD) age, years	48.21 (18.57)	80.27 (12.10)	69.63 (17.91)
Age band:			
19-29 years	1 139 120 (18.73)	12 (0.27)	282 (2.62)
30-39 years	1 190 905 (19.58)	22 (0.50)	523 (4.85)
40-49 years	1 021 643 (16.79)	51 (1.16)	828 (7.68)
50-59 years	1 013 599 (16.66)	223 (5.09)	1371 (12.72)
60-69 years	757 483 (12.45)	460 (10.49)	1677 (15.56)
70-79 years	586 164 (9.64)	892 (20.35)	2135 (19.81)
80-89 years	298 093 (4.90)	1722 (39.28)	2722 (25.26)
≥90 years	76 095 (1.25)	1002 (22.86)	1238 (11.49)
Geographical region:			
East Midlands	164 502 (2.70)	52 (1.19)	131 (1.22)
East of England	186 673 (3.07)	136 (3.10)	317 (2.94)
London	1 576 616 (25.92)	1287 (29.36)	3799 (35.25)
North East	163 388 (2.69)	87 (1.98)	243 (2.26)
North West	1 076 590 (17.70)	942 (21.49)	2055 (19.07)
South Central	824 558 (13.55)	563 (12.84)	1293 (12.00)
South East	685 960 (11.28)	462 (10.54)	960 (8.91)
South West	581 014 (9.55)	198 (4.52)	501 (4.65)
West Midlands	605 752 (9.96)	533 (12.16)	1197 (11.11)
Yorkshire and Humber	218 049 (3.58)	124 (2.83)	280 (2.60)
Ethnicity:			
White	3 924 110 (64.51)	2947 (67.22)	6790 (63.01)
Indian	175 909 (2.89)	131 (2.99)	423 (3.93)
Pakistani	114 727 (1.89)	69 (1.57)	248 (2.30)
Bangladeshi	87 491 (1.44)	69 (1.57)	173 (1.61)
Other Asian	110 579 (1.82)	57 (1.30)	248 (2.30)
Caribbean	69 166 (1.14)	152 (3.47)	392 (3.64)
Black African	150 022 (2.47)	122 (2.78)	456 (4.23)
Chinese	58 511 (0.96)	18 (0.41)	45 (0.42)
Other ethnic group	224 394 (3.69)	114 (2.60)	436 (4.05)
Not recorded	1 168 193 (19.20)	705 (16.08)	1565 (14.52)
Townsend deprivation fifth:			
1 (most affluent)	1 238 575 (20.36)	840 (19.16)	1799 (16.69)
2	1 222 681 (20.10)	746 (17.02)	1886 (17.50)
3	1 187 082 (19.51)	934 (21.30)	2114 (19.62)
4	1 176 829 (19.35)	951 (21.69)	2338 (21.70)
5 (most deprived)	1 23 1431 (20.24)	905 (20.64)	2612 (24.24)
Not recorded	26 504 (0.44)	*	27 (0.25)
Accommodation:			
Neither homeless nor care home resident	6 036 288 (99.23)	3345 (76.30)	9895 (91.82)
Care home or nursing home resident	35 813 (0.59)	1033 (23.56)	854 (7.93)
Homeless	11 001 (0.18)	*	27 (0.25)
Body mass index:			
<18.5	161 579 (2.66)	203 (4.63)	260 (2.41)
18.5-24.99	2 033 809 (33.43)	1345 (30.68)	2708 (25.13)
25-29.99	1 723 494 (28.33)	1291 (29.45)	3406 (31.61)
30-34.99	800 857 (13.17)	738 (16.83)	2126 (19.73)
≥35	453 323 (7.45)	460 (10.49)	1549 (14.37)
Not recorded	910 040 (14.96)	347 (7.92)	727 (6.75)
Smoking status:			
Non-smoker	3 482 456 (57.25)	2312 (52.74)	6073 (56.36)
Ex-smoker	1 291 953 (21.24)	1735 (39.58)	3716 (34.48)
Light smoker	803 783 (13.21)	199 (4.54)	668 (6.20)
Moderate smoker	153 680 (2.53)	32 (0.73)	97 (0.90)
Heavy smoker	70 215 (1.15)	18 (0.41)	62 (0.58)
Not recorded	281 015 (4.62)	88 (2.01)	160 (1.48)
Chronic kidney disease (CKD):			
No CKD	5 843 919 (96.07)	2928 (66.79)	8156 (75.69)
CKD3	2 14193 (3.52)	1190 (27.14)	2010 (18.65)
CKD4	12 654 (0.21)	141 (3.22)	252 (2.34)
CKD5 only	7286 (0.12)	96 (2.19)	239 (2.22)
CKD5 with dialysis	1676 (0.03)	14 (0.32)	46 (0.43)
CKD5 with transplant	3374 (0.06)	15 (0.34)	73 (0.68)
Learning disability:			
No learning disability	5 972 982 (98.19)	4110 (93.75)	10251 (95.13)
Learning disability	107107 (1.76)	255 (5.82)	498 (4.62)
Down’s syndrome	3013 (0.05)	19 (0.43)	27 (0.25)
Chemotherapy:			
No chemotherapy in previous 12 months	6 059 236 (99.61)	4267 (97.33)	10482 (97.27)
Chemotherapy group A	9307 (0.15)	33 (0.75)	71 (0.66)
Chemotherapy group B	13 600 (0.22)	75 (1.71)	200 (1.86)
Chemotherapy group C	959 (0.02)	*	23 (0.21)
Cancer and immunosuppression:			
Blood cancer	28 089 (0.46)	114 (2.60)	238 (2.21)
Bone marrow or stem cell transplant in previous 6 months	194 (0.00)	*	*
Respiratory cancer	12 792 (0.21)	61 (1.39)	130 (1.21)
Radiotherapy in previous 6 months	12 129 (0.20)	56 (1.28)	125 (1.16)
Solid organ transplant	3209 (0.05)	10 (0.23)	33 (0.31)
GP prescribed immunosuppressant medication	7990 (0.13)	19 (0.43)	53 (0.49)
Prescribed leukotriene or LABA	13 0895 (2.15)	399 (9.10)	874 (8.11)
Prescribed regular prednisolone	32 929 (0.54)	176 (4.01)	388 (3.60)
Sickle cell disease	2125 (0.03)	*	28 (0.26)
Other comorbidities:			
Type 1 diabetes	28 587 (0.47)	36 (0.82)	136 (1.26)
Type 2 diabetes	394 562 (6.49)	1417 (32.32)	3017 (28.00)
Chronic obstructive pulmonary disease	142 107 (2.34)	580 (13.23)	1155 (10.72)
Asthma	825 422 (13.57)	584 (13.32)	1745 (16.19)
Rare pulmonary diseases	33 433 (0.55)	96 (2.19)	240 (2.23)
Pulmonary hypertension or pulmonary fibrosis	4940 (0.08)	40 (0.91)	83 (0.77)
Coronary heart disease	215 069 (3.54)	1038 (23.68)	1779 (16.51)
Stroke	129 699 (2.13)	809 (18.45)	1339 (12.43)
Atrial fibrillation	147 528 (2.43)	832 (18.98)	1461 (13.56)
Congestive cardiac failure	70 970 (1.17)	575 (13.12)	1005 (9.33)
Venous thromboembolism	105 136 (1.73)	381 (8.69)	753 (6.99)
Peripheral vascular disease	44 476 (0.73)	289 (6.59)	467 (4.33)
Congenital heart disease	31 576 (0.52)	48 (1.09)	100 (0.93)
Dementia	58 873 (0.97)	1311 (29.90)	1235 (11.46)
Parkinson’s disease	15 315 (0.25)	137 (3.13)	218 (2.02)
Epilepsy	80 064 (1.32)	159 (3.63)	348 (3.23)
Rare neurological conditions	18 603 (0.31)	42 (0.96)	120 (1.11)
Cerebral palsy	6481 (0.11)	*	27 (0.25)
Severe mental illness	672 494 (11.06)	745 (16.99)	1841 (17.08)
Osteoporotic fracture	238 276 (3.92)	675 (15.40)	1154 (10.71)
Rheumatoid arthritis or SLE	60 847 (1.00)	127 (2.90)	309 (2.87)
Cirrhosis of liver	11 865 (0.20)	37 (0.84)	106 (0.98)

GP=general practitioner; LABA=long acting β agonist; SLE=systemic lupus erythematosus.

* Value suppressed owing to small numbers (<15).

In the derivation cohort, 10 776 (0.18%) patients had a covid-19 related hospital admission and 4384 (0.07%) had a covid-19 related death during the 97 days’ follow-up, of which 4265 (97.3%) were recorded on the death certificate and 119 (2.71%) were based only on a positive test (and of these <15 were based on a test more than 28 days before death). Admissions and deaths due to covid-19 occurred across all regions, with the greatest numbers in London, which accounted for 3799 (35.3%) of admissions and 1287 (29.4%) of deaths. Of those who died, 2517 (57.4%) were male, 732 (16.7%) were BAME, 3616 (82.5%) were aged 70 and over, 1417 (32.3%) had type 2 diabetes, 1311 (29.9%) had dementia, and 1033 (23.6%) were identified as living in a care home.

The characteristics of the validation cohort were similar to those of the derivation cohort, as shown in supplementary tables A and B. In the first validation period (24 January to 30 April 2020), 1722 deaths and 3703 hospital admissions due to covid-19 occurred. In the second validation period (1 May to 30 June 2020), 621 deaths and 1002 admissions due to covid-19 occurred.

### Predictor variables

**Fig 1:**
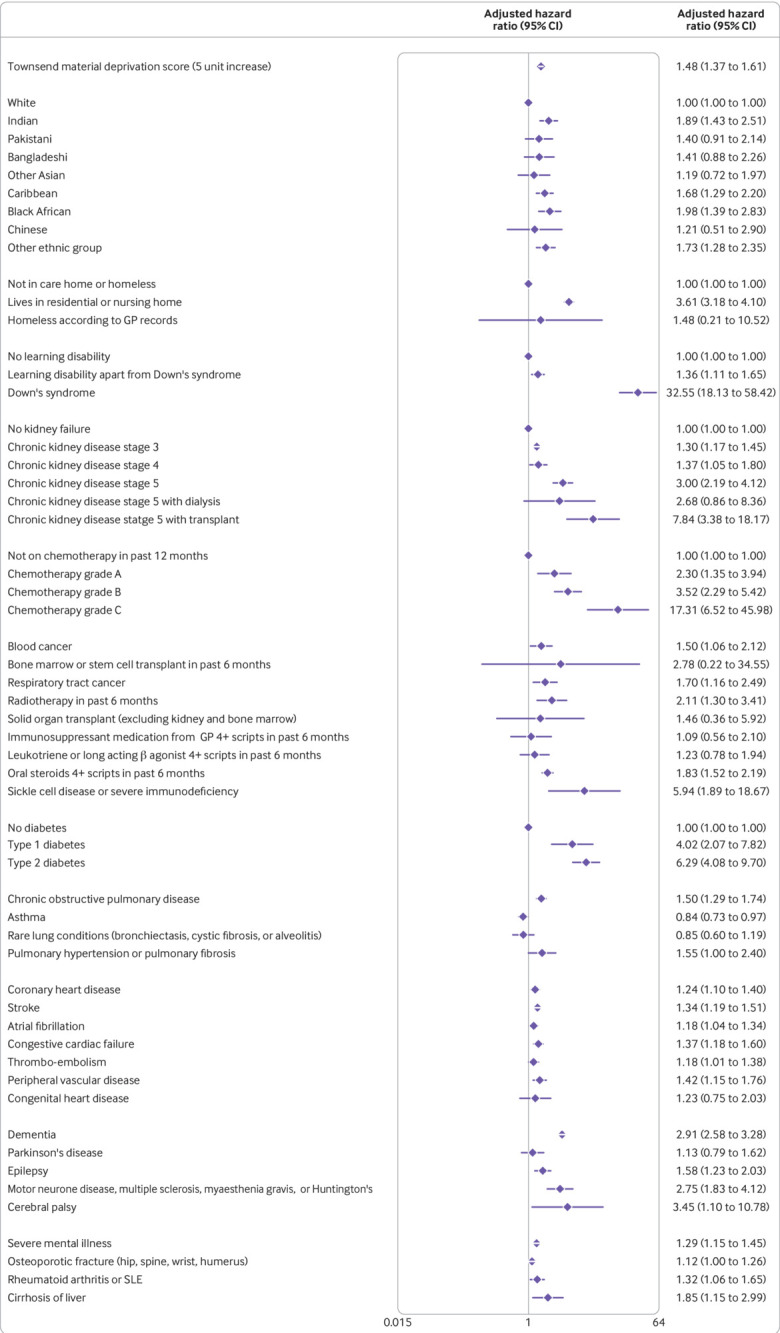
Adjusted hazard ratio (95% CI) of death from covid-19 in women in derivation cohort, adjusted for variables shown, deprivation, and fractional polynomial terms for body mass index (BMI) and age. Model includes fractional polynomial terms for age (3 3) and BMI (0.5 0.5 ln(bmi)) and interaction terms between age terms and type 2 diabetes. Hazard ratio for type 2 diabetes reported at mean age. GP=general practitioner; SLE=systemic lupus erythematosus. (QResearch database version 44; study period 24 January 2020 to 30 April 2020)

**Fig 2:**
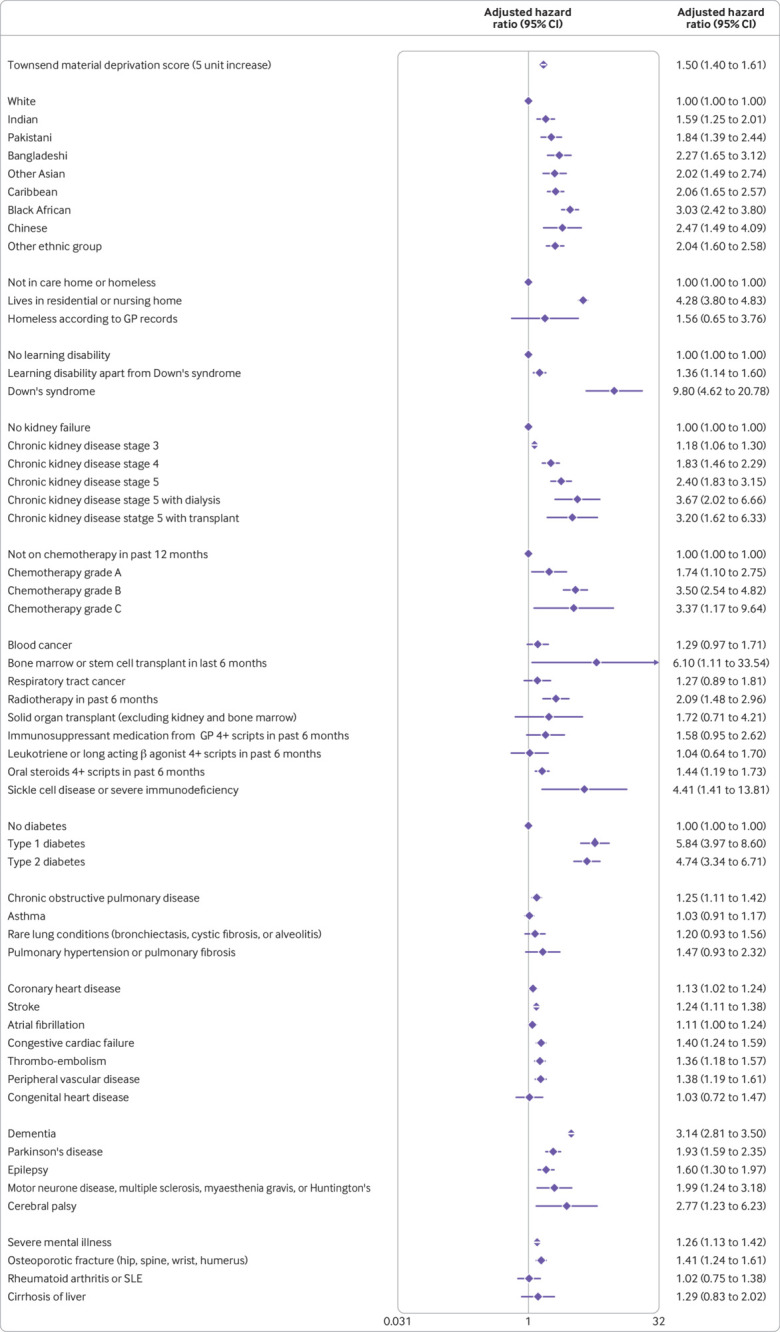
Adjusted hazard ratio (95% CI) of death from covid-19 in men in derivation cohort, adjusted for variables shown, deprivation, and fractional polynomial terms for body mass index (BMI) and age. Model includes fractional polynomial terms for age (1 3) and BMI (−0.5 −0.5 ln(age)) and interaction terms between age terms and type 2 diabetes. Hazard ratio for type 2 diabetes reported at mean age. GP=general practitioner; SLE=systemic lupus erythematosus. (QResearch database version 44; study period 24 January 2020 to 30 April 2020)

**Fig 3:**
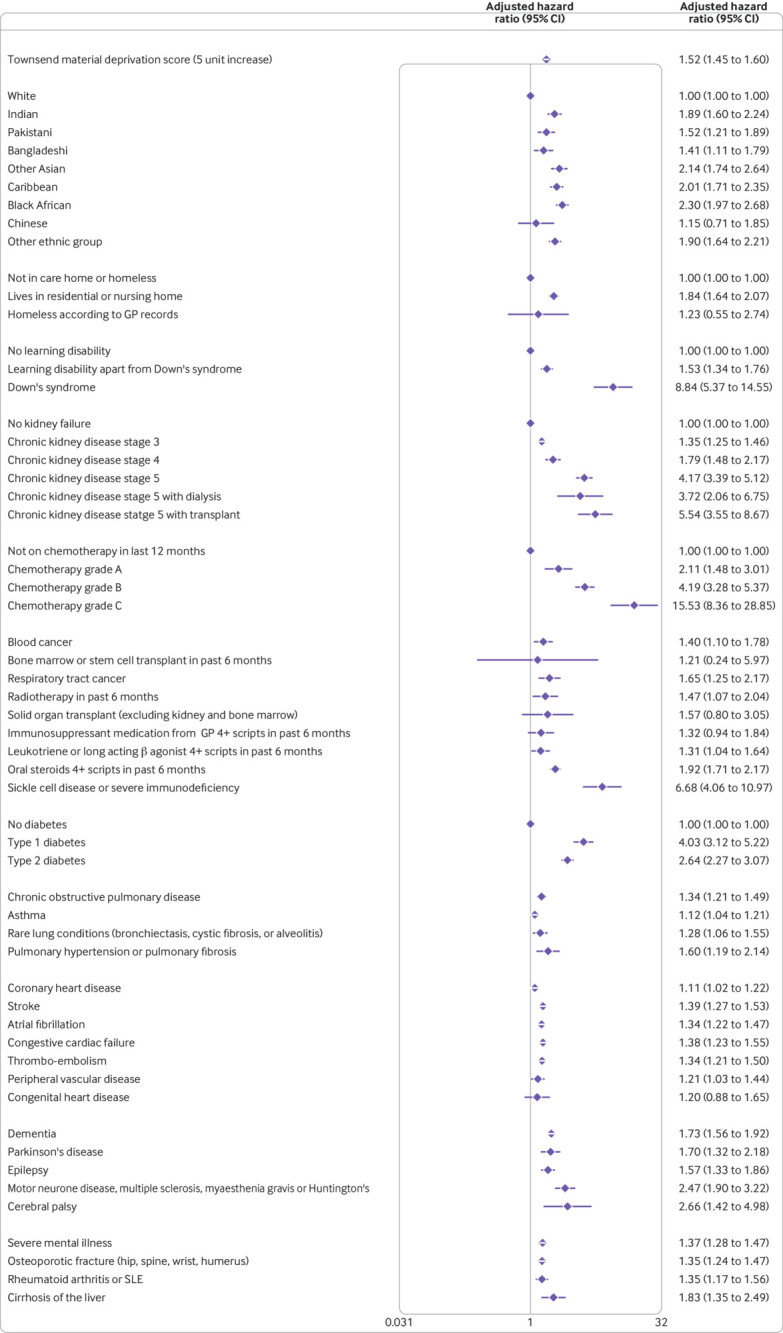
Adjusted hazard ratio (95%CI) of hospital admission for covid-19 in women in derivation cohort, adjusted for variables shown, deprivation, fractional polynomial terms for body mass index (BMI) and age. Model includes fractional polynomial terms for age (0.5, 2) and BMI (−2 0) and interaction terms between age terms and type 2 diabetes. Hazard ratio for type 2 diabetes reported at mean age. GP=general practitioner; SLE=systemic lupus erythematosus. (QResearch database version 44; study period 24 January 2020 to 30 April 2020)

**Fig 4:**
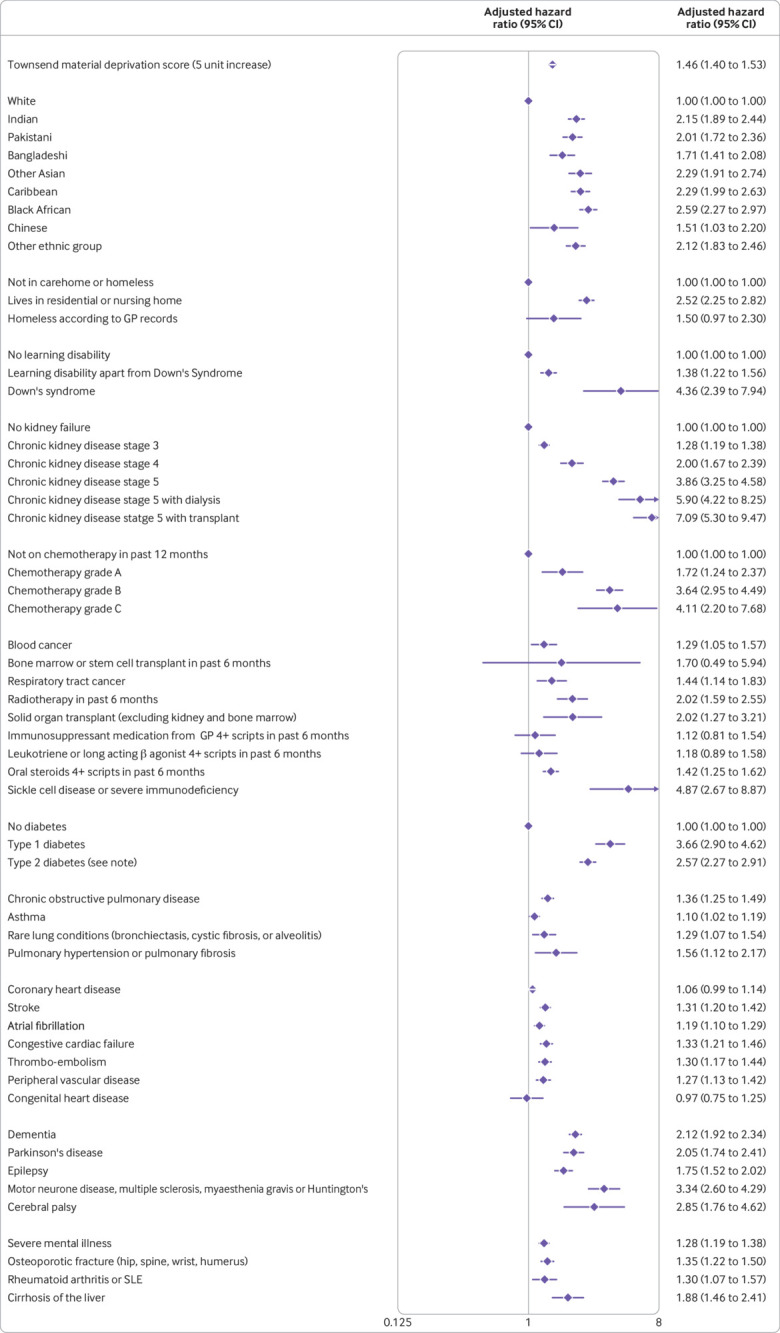
Adjusted hazard ratio (95% CI) of hospital admission for covid-19 in men in derivation cohort, adjusted for variables shown, deprivation, and fractional polynomial terms for body mass index (BMI) and age. Model includes fractional polynomial terms for age (−2 2) and BMI (−0.5 0) and interaction terms between age terms and type 2 diabetes. Hazard ratio for type 2 diabetes reported at mean age. GP=general practitioner; SLE=systemic lupus erythematosus. (QResearch database version 44; study period 24 January 2020 to 30 April 2020)

The variables included in the final models were fractional polynomial terms for age and body mass index, Townsend score (linear), ethnic group, domicile (residential care, homeless, neither), and a range of conditions and treatments as shown in figure 1, figure 2, figure 3, and figure 4. These conditions and treatments were cardiovascular conditions (atrial fibrillation, heart failure, stroke, peripheral vascular disease, coronary heart disease, congenital heart disease), diabetes (type 1 and type 2 and interaction terms for type 2 diabetes with age), respiratory conditions (asthma, rare respiratory conditions (cystic fibrosis, bronchiectasis, or alveolitis), chronic obstructive pulmonary disease, pulmonary hypertension or pulmonary fibrosis), cancer (blood cancer, chemotherapy, lung or oral cancer, marrow transplant, radiotherapy), neurological conditions (cerebral palsy, Parkinson’s disease, rare neurological conditions (motor neurone disease, multiple sclerosis, myasthenia, Huntington’s chorea), epilepsy, dementia, learning disability, severe mental illness), other conditions (liver cirrhosis, osteoporotic fracture, rheumatoid arthritis or systemic lupus erythematosus, sickle cell disease, venous thromboembolism, solid organ transplant, renal failure (CKD3, CKD4, CKD5, with or without dialysis or transplant)), and medications (≥4 prescriptions from general practitioner in previous six months for oral steroids, long acting β agonists or leukotrienes, immunosuppressants).

Figure 1 and figure 2 show the adjusted hazard ratios in the final models for covid-19 related death in the derivation cohort in women and men. Figure 3 and figure 4 show the adjusted hazard ratios for the final models for covid-19 related hospital admission in the derivation cohort.

Supplementary figures A and B show graphs of the adjusted hazard ratios for body mass index, age, and the interaction between age and type 2 diabetes for deaths and hospital admissions due to covid-19 (which showed higher risks associated with younger ages). Supplementary figures C and D show fully adjusted hazard ratios for variables for the full model, including variables that were not retained in the final model (for example, adjusted hazard ratios close to one or those which lacked clinical credibility). Other variables with too few events for inclusion were HIV, sphingolipidoises, short bowel syndrome, polymyositis, dermatomyositis, Ehlers-Danlos syndrome, biliary cirrhosis, hepatitis B and C, haemochromatosis, non-alcoholic fatty liver disease, chronic pancreatitis, drug misuse, asplenia, cholangitis, scleroderma, Sjogren’s syndrome, and pregnancy. Supplementary figures E and F show fully adjusted hazard ratios for a combined outcome of either covid-19 related death or hospital admission. This gave very similar absolute risks to the hospital admission outcome.

### Model evaluation

#### Discrimination

Table 3 shows the performance of the risk equations in the validation cohort for women and men over 97 days for the main study period and for the temporal validation cohort evaluated from 1 May 2020 to 30 June 2020. Overall, the values for the R^2^, D, and C statistics were similar in women and men. Values for the mortality outcome tended to be higher than those for the hospital admission outcome. For example, in the first validation period, the equation explained 74% of the variation in time to death from covid-19 in women; the D statistic was 3.46, and Harrell’s C statistic was 0.933. The corresponding values in men were 73.1%, 3.37, and 0.928. The results for the second validation period were similar except for covid-19 related admissions in women, for which the explained variation and discrimination were lower than for the first period (explained variation 45.4%, D statistic 1.87, and Harrell’s C statistic 0.776).

**Table 3:** Performance of risk models to predict risk of death and hospital admission due to covid-19 in validation cohort in first validation period (24 January to 30 April 2020) and second temporal validation (1 May to 30 June 2020). Values are estimates (95% CIs) unless stated otherwise

	Covid-19 death			Covid-19 admission	
	Women	Men		Women	Men
**Period 1**					
R^2^ statistic (%)	74.0 (72.7 to 75.3)	73.1 (71.9 to 74.3)		57.1 (55.5 to 58.8)	58.1 (56.7 to 59.5)
D statistic	3.46 (3.34 to 3.57)	3.37 (3.27 to 3.47)		2.36 (2.28 to 2.44)	2.41 (2.34 to 2.48)
Harrell’s C	0.933 (0.923 to 0.944)	0.928 (0.919 to 0.938)		0.847 (0.836 to 0.857)	0.860 (0.852 to 0.868)
Brier score	0.0007	0.0009		0.0015	0.0019
**Period 2**					
R^2^ statistic (%)	75.4 (73.5 to 77.4)	73.6 (71.6 to 75.6)		45.4 (41.7 to 49.1)	55.4 (52.2 to 58.5)
D statistic	3.59 (3.4 to 3.77)	3.42 (3.24 to 3.59)		1.87 (1.73 to 2)	2.28 (2.14 to 2.42)
Harrell’s C	0.952 (0.938 to 0.965)	0.933 (0.918 to 0.949)		0.776 (0.753 to 0.800)	0.833 (0.812 to 0.853)
Brier score	0.0002	0.0003		0.0004	0.0004

Supplementary tables C-F show the corresponding results by region, age band, and fifth of deprivation and within each ethnic group in men and women in both validation periods. Performance was generally similar to the overall results except for age, for which the values were lower within individual age bands.

**Fig 5:**
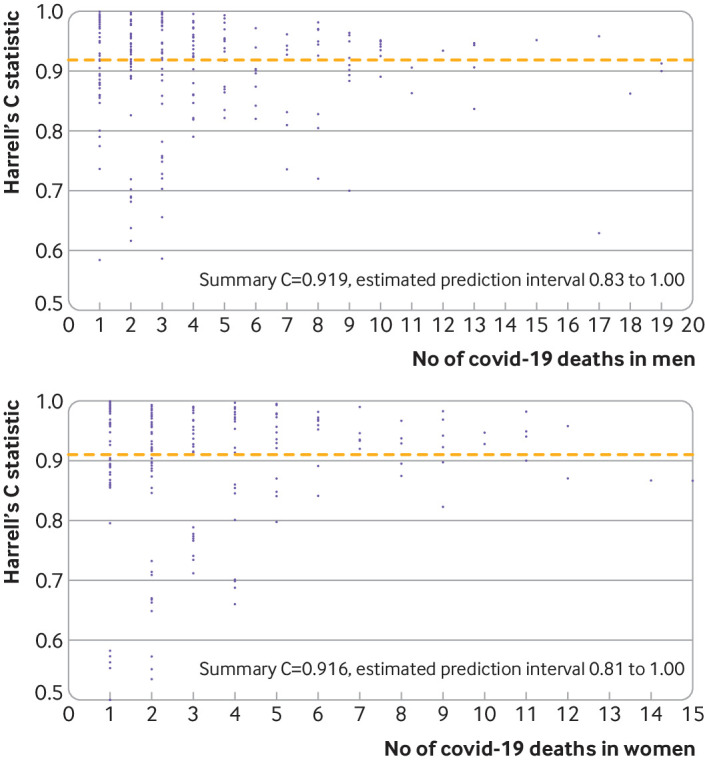
Funnel plots of discrimination using Harrell’s C statistic for each general practice in validation cohort versus number of deaths in each practice in men (top panel) and women (bottom panel) in first validation period

Figure 5 shows funnel plots of Harrell’s C statistic for each general practice in the validation cohort versus the number of deaths in each practice in men and women in the first validation period. The summary (average) C statistic for women was 0.916 (95% confidence interval 0.908 to 0.924) from a random effects meta-analysis. The corresponding summary C statistic for men was 0.919 (0.912 to 0.926).

#### Calibration

**Fig 6:**
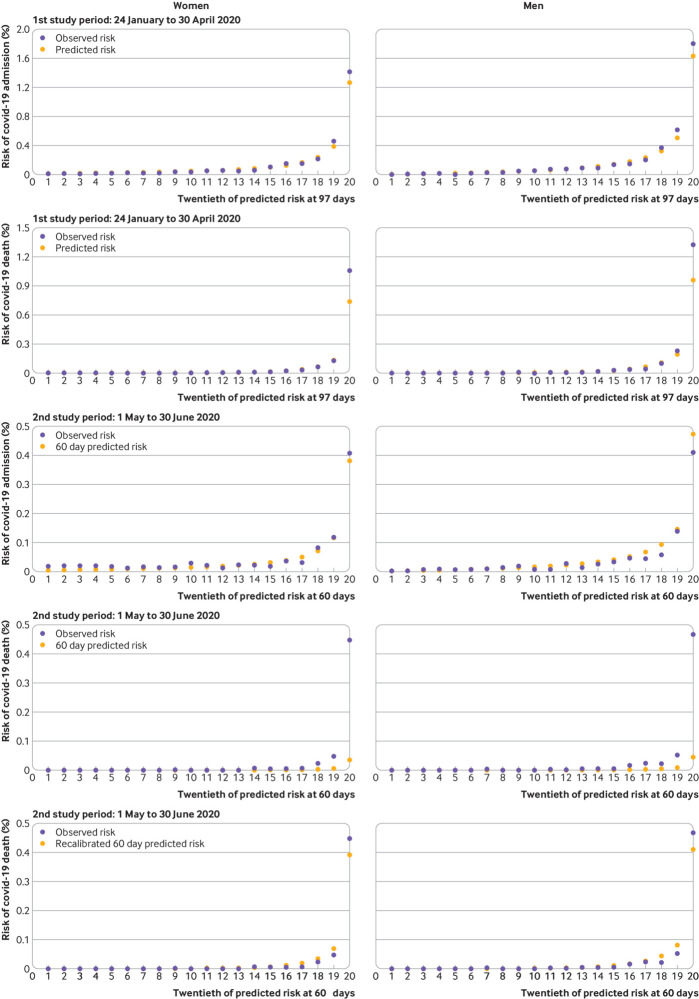
Predicted and observed risk of covid-19 related hospital admission and death in validation cohort in first study period (24 January to 30 April 2020) and in second study period (1 May to 30 June 2020), and recalibrated predicted and observed risk of covid-19 related death in validation cohort in second study period (1 May to 30 June 2020)

Figure 6 (top two rows) shows the calibration plots for the covid-19 related hospital admission equation and for the covid-19 related death equation in the first validation period. These show that both sets of equations were well calibrated in the first time period except for a small degree of under-prediction in the highest risk category for mortality. In the second validation period, calibration was good for the hospital admission outcome but not for the mortality outcome at the high levels of risk (fig 6, third and fourth rows). The calibration was improved with recalibration, with observed risks more closely matching the predicted risks (fig 6, bottom row).

#### Risk stratification

Table 4 shows the sensitivity values for the mortality equation over 97 days evaluated at different thresholds based on the centiles of the predicted absolute risk in the validation cohort. For example, it shows that 75.73% of deaths occurred in people in the top 5% for predicted absolute risk of death from covid-19 (predicted absolute risks above 0.237%). People in the top 20% for predicted absolute risk of death accounted for 94% of deaths, and the top 25% accounted for 95.99% of deaths. Supplementary table G shows a similar table based on centiles of relative risk. This example shows that 50.93% of deaths occurred in people in the top 5% for predicted relative risk of covid-19 related death (predicted relative risk above 5.3). People in the top 20% for predicted relative risk of death accounted for 77.53% of deaths, and the top 25% accounted for 81.59% of deaths. As an example, table 5 shows characteristics of the 5% of patients with the highest predicted absolute risk of death for all individuals aged 19-100 years.

**Table 4:** Sensitivity for covid-19 related death over 97 days in validation cohort (24 January to 30 April 2020) comprising 2 173 056 patients with 1722 covid-19 related deaths at different absolute risk thresholds*

Top centile	Total patients in each centile	Absolute risk centile cut-off (%)	Total deaths in each absolute risk centile	Cumulative % deaths based on absolute risk (sensitivity†)
1	21730	0.9093	708	41.11
2	21731	0.5182	263	56.39
3	21730	0.3703	136	64.29
4	21731	0.2892	105	70.38
5	21730	0.2369	92	75.73
6	21731	0.1990	58	79.09
7	21730	0.1702	35	81.13
8	21731	0.1473	46	83.80
9	21731	0.1288	26	85.31
10	21730	0.1135	24	86.70
11	21731	0.1004	18	87.75
12	21730	0.0895	19	88.85
13	21731	0.0800	19	89.95
14	21730	0.0719	18	91.00
15	21731	0.0647	7	91.41
16	21730	0.0584	5	91.70
17	21731	0.0528	14	92.51
18	21731	0.0477	12	93.21
19	21730	0.0432	9	93.73
20	21731	0.0393	5	94.02
21	21730	0.0357	6	94.37
22	21731	0.0325	9	94.89
23	21730	0.0296	6	95.24
24	21731	0.0270	4	95.47
25	21731	0.0246	9	95.99

* Centile value giving cut-off of predicted risk over 97 days for defining each group of absolute risk.

† Percentage of total deaths over 97 days that occurred within group of patients above predicted risk threshold.

**Table 5:** Summary characteristics for top 5% of patients with highest predicted absolute risks of covid-19 death. Table shows results for all members of validation cohort

Characteristic	Total population (n=2 173 056)	Total (column %) in top 5% predicted risk (n=108 652)	Top 5% predicted risk (row %)
Male sex	1 075 788	63 755 (58.68)	5.93
Age band:			
19-29 years	424 125	*	*
30-39 years	417 590	*	*
40-49 years	358 695	97 (0.09)	0.03
50-59 years	358 820	1028 (0.95)	0.29
60-69 years	270 340	6428 (5.92)	2.38
70-79 years	209 557	25 542 (23.51)	12.19
≥80 years	133 929	75 547 (69.53)	56.41
Ethnicity:			
White	1 780 507	90 958 (83.71)	5.11
Indian	64 184	3034 (2.79)	4.73
Pakistani	40 718	1863 (1.71)	4.58
Bangladeshi	28 050	1247 (1.15)	4.45
Other Asian	42 607	1489 (1.37)	3.49
Caribbean	28 741	3702 (3.41)	12.88
Black African	58 115	2884 (2.65)	4.96
Chinese	29 972	603 (0.55)	2.01
Other ethnic group	100 162	2872 (2.64)	2.87
Townsend deprivation fifth:			
1 (most affluent)	446 359	20 010 (18.42)	4.48
2	428 735	20 524 (18.89)	4.79
3	439 846	23 758 (21.87)	5.40
4	436 574	23 644 (21.76)	5.42
5 (most deprived)	409 917	20 437 (18.81)	4.99
Townsend not recorded	11 625	279 (0.26)	2.40
Accommodation:			
Neither homeless or care home resident	2 155 199	97 210 (89.47)	4.51
Care home or nursing home resident	14 057	11 269 (10.37)	80.17
Homeless	3800	173 (0.16)	4.55
Body mass index:			
<18.5	59 376	4188 (3.85)	7.05
18.5-24.99	711 186	33 122 (30.48)	4.66
25-29.99	596 942	34 044 (31.33)	5.70
30-34.99	278 830	18 762 (17.27)	6.73
≥35	160 345	13 086 (12.04)	8.16
Not recorded	366 377	5450 (5.02)	1.49
Chronic kidney disease (CKD)			
No CKD	2 087 614	68 710 (63.24)	3.29
CKD3	76 600	34 418 (31.68)	44.93
CKD4	4648	3194 (2.94)	68.72
CKD5 only	2527	1722 (1.58)	68.14
CKD5 with dialysis	585	274 (0.25)	46.84
CKD5 with transplant	1082	334 (0.31)	30.87
Learning disability:			
No learning disability	2 137 759	103 919 (95.64)	4.86
Learning disability	34 257	4473 (4.12)	13.06
Down’s syndrome	1040	260 (0.24)	25.00
Chemotherapy:			
No chemotherapy in previous 12 months	2 164 341	105 131 (96.76)	4.86
Chemotherapy group A	3343	1100 (1.01)	32.90
Chemotherapy group B	5032	2223 (2.05)	44.18
Chemotherapy group C	340	198 (0.18)	58.24
Cancer and immunosuppression:			
Blood cancer	10 359	3084 (2.84)	29.77
Bone marrow or stem cell transplant in previous 6 months	73	56 (0.05)	76.71
Respiratory cancer	4549	1722 (1.58)	37.85
Radiotherapy in previous 6 months	4346	1709 (1.57)	39.32
Solid organ transplant	1147	283 (0.26)	24.67
GP prescribed immunosuppressant medication	2814	455 (0.42)	16.17
Prescribed leukotriene or LABA	45 905	9591 (8.83)	20.89
Prescribed regular prednisolone	11 617	4518 (4.16)	38.89
Sickle cell disease	717	117 (0.11)	16.32
Other comorbidities:			
Type 1 diabetes	10 337	861 (0.79)	8.33
Type 2 diabetes	137 092	40 674 (37.44)	29.67
Chronic obstructive pulmonary disease	51 026	16 708 (15.38)	32.74
Asthma	299 632	14 860 (13.68)	4.96
Rare pulmonary diseases	11 748	2868 (2.64)	24.41
Pulmonary hypertension or pulmonary fibrosis	1891	1061 (0.98)	56.11
Coronary heart disease	77 035	29 476 (27.13)	38.26
Stroke	47 513	20 384 (18.76)	42.90
Atrial fibrillation	52 764	23 579 (21.70)	44.69
Congestive cardiac failure	25 255	14 897 (13.71)	58.99
Venous thromboembolism	38 962	10114 (9.31)	25.96
Peripheral vascular disease	16 463	8005 (7.37)	48.62
Congenital heart disease	11 344	1288 (1.19)	11.35
Dementia	21 984	19 829 (18.25)	90.20
Parkinson’s disease	5736	2847 (2.62)	49.63
Epilepsy	29 031	3503 (3.22)	12.07
Rare neurological conditions	6804	1092 (1.01)	16.05
Cerebral palsy	2433	233 (0.21)	9.58
Severe mental illness	246 668	17 428 (16.04)	7.07
Osteoporotic fracture	87 595	15 933 (14.66)	18.19
Rheumatoid arthritis or SLE	21 391	3251 (2.99)	15.20
Cirrhosis of liver	4442	1054 (0.97)	23.73

GP=general practitioner; LABA=long acting β agonist; SLE=systemic lupus erythematosus.

* Values suppressed owing to small numbers <15.

Supplementary figures G and H show two clinical examples from the web calculator (https://qcovid.org/BMJ/), showing the absolute and relative risk of catching and dying from covid-19 and the risk of hospital admission due to covid-19. It also shows a ranking of mortality risk based on centiles across the validation cohort. Supplementary figure G shows a 55 year old black African man with type 2 diabetes, a body mass index of 27.7, and no other risk factors. His absolute risk of catching and dying from covid-19 over the 90 day period is 0.1095% (or 1 in 913). His relative risk compared with a white man aged 55 years and a body mass index of 25 is 10.84. The graph shows that he is in the top 10% of the population at highest risk. Supplementary figure H shows a 30 year old white woman with Down’s syndrome with a body mass index of 40. Her absolute risk of catching and dying from covid-19 is 0.024% (or 1 in 4184). Her relative risk compared with a white woman aged 30 years with a body mass index of 25 and no other risk factors is 59.75, and the rank is 75. Her absolute risk of admission to hospital with covid-19 over 90 days is 1 in 272.

## Discussion

We have developed and evaluated a novel clinical risk prediction model (QCOVID) to estimate risks of hospital admission and mortality due to covid-19. We have used national linked datasets from general practice and national SARS-CoV-2 testing, death registry, and hospital episode data for a sample of more than 8 million adults representative of the population of England. The risk models have excellent discrimination (Harrell’s C statistics >0.9 for the primary outcome). Although the calibration for the hospital admission outcome was good in both time periods, some under-prediction existed for the mortality outcome in the second validation cohort, which improved after recalibration. The recalibration method could be used to transport the risk models to other settings or time periods with different absolute risks of covid-19. QCOVID represents a new approach for risk stratification in the population. It could also be deployed in several health and care applications, either during the current phase of the pandemic or in subsequent “waves” of infection (with recalibration as needed). These could include supporting targeted recruitment for clinical trials, prioritisation for vaccination, and discussions between patients and clinicians on workplace or health risk mitigation—for example, through weight reduction as obesity may be an important modifiable risk factor for serious complications of covid-19 if a causal association is established.^10^ Although QCOVID has been specifically designed to inform UK health policy and interventions to manage covid-19 related risks, it also has international potential, subject to local validation. One of the variables in our model (the Townsend measure of deprivation) may need to be replaced with locally available equivalent measures, or some recalibration may be needed. Previous risk prediction models based on QResearch have been validated internationally and found to have good performance outside of the UK.^29^ ^30^

### Comparison with other studies

Although similarities exist between our study and the recently reported analysis of risk factors from another English general practice database using a different clinical computer system, our project had a different aim—namely, to develop and evaluate a risk prediction model. We used a more comprehensive outcome (including deaths in patients with positive tests for SARS-CoV-2), a much wider range of predictors, and a more granular assessment of ethnicity and body mass index. Our C statistic for mortality (>0.92) is substantially higher than the previous study’s reported value of 0.77.^31^ Other prediction models have been reported, although these focus on other outcomes of covid-19, including risk of admission to intensive care or death following a positive test, or clinical decision tools that integrate biochemical and imaging parameters to aid diagnostis.^13^ However, most such studies are at high risk of bias, as they have been developed in highly selected cohorts, have limited transparency, are likely to have optimistic reported performance, or did not use covid-19 specific data.^13^ This study represents a substantial improvement on previously developed risk algorithms in terms of the size and representativeness of the study population, the richness of data linkages enabling accurate ascertainment of cases (including both in-hospital and out of hospital deaths) across the health network, and the breadth of candidate predictor variables considered. Importantly, it analyses risks at the population level, rather than risks in people with confirmed or suspected infection, and may have relevance for shielding or other policies that seek to mitigate risk of viral exposure.

### Complexities of modelling 

Several complexities of modelling adverse risks from covid-19 in the general population warrant discussion. We used a general population approach which, although not able to incorporate all determinants of being infected, offers an overall estimate of risk of adverse outcomes from covid-19 that could be used in discussions between clinicians and patients about adjustment of lifestyle or occupational and behavioural factors that could limit viral exposure. Our model predicts risks of “catching covid-19 and then having a severe outcome,” on the basis of data collected during the first peak of the pandemic. The endpoint in this study examines a risk trajectory that comprises two elements: becoming infected, which is predominantly a function of behavioural/environmental factors including occupation, local infection rate, and numbers of social interactions; and risk of hospital admission and death due to the infection, which is arguably primarily driven by “vulnerability” (that is, biological/physiological factors including age, sex, body mass index, comorbidities, and medications). Although producing a prediction model for risk of “death if infected” is feasible in principle, this approach is not yet possible owing to the approach to testing in the UK and the context of an as yet incompletely quantified degree of asymptomatic background transmission. Limited covid-19 testing data are available, but the difficulty is that no systematic community testing was done in the UK during the study period, so only patients unwell enough to attend hospital were tested. This means that a risk score developed in those who tested positive would overestimate risks of severe outcomes. As more widespread testing is done and those data become available, we will be able to update the model to take background infection rates into account and also model regional differences. Although the absolute risk levels will of course change over time, depending on the incidence of the disease, our analysis over two validation time periods indicates that the relative risk measures and discrimination are likely to remain stable.

Secondly, the model estimates the absolute risk for a non-infected individual in the general population of becoming infected and then dying (or needing to be admitted to hospital) from the virus over a 97 day period. Although many more than 40 000 people have died from covid-19 in the UK to date, when the denominator is a population of multi-millions, the absolute risk for most people may be low. Therefore, when conveying this type of risk score to an individual, due emphasis is needed on the different meanings of absolute and relative risk.

Thirdly, the absolute risk of catching covid-19 depends not only on the incidence of the infection but also on the number of people one gets close to. For this reason, non-pharmacological interventions such as social distancing and shielding were introduced in the UK during the study period. We have included some measures of multi-occupancy, as we have factored care homes into the analysis. The data generated during the study period will therefore be affected by the uptake of interventions such as social distancing and shielding, intended to mitigate the risks of SARS-CoV-2 infection. This could result in underestimation of some model coefficients and hence underestimation of absolute risk in people who were shielded. Also, as this is a prediction model derived from an observational study, the associations estimated for individual predictor variables should not be interpreted as causal effects.

However, ethical questions must be considered regarding how the tools may be used. We have presented two ways of stratifying risk based on either absolute or relative risk measures with associated centile values, but the choice of whether to have a threshold (given that risk is a continuous measure), and if so what threshold, will depend on the purpose for which the risk assessment tool is to be used, the available resources, and the ethical framework for decision making. We have analysed this within the “four ethical principles” framework that is widely used in medical decision making. The four principles are autonomy, beneficence, justice, and non-maleficence.^32^ The new risk equations, when implemented in clinical software, are designed to provide more accurate information for patients and clinicians on which to base decisions, thereby promoting shared decision making and patient autonomy. They are intended to result in clinical benefit by identifying where changes in management are likely to benefit patients, thereby promoting the principle of beneficence. Justice can be achieved by ensuring that the use of the risk equations results in fair and equitable access to health services that is commensurate with patients’ level of risk. Lastly, the risk assessment must not be used in a way that causes harm either to the individual patient or to others (for example, by introducing or withdrawing treatments where this is not in the patient’s best interest), thereby supporting the non-maleficence principle. How this applies in clinical practice will naturally depend on many factors, especially the patient’s wishes, the evidence base for any interventions, the clinician’s experience, national priorities, and the available resources. The risk assessment equations therefore supplement clinical decision making and do not replace it. With these caveats, the predicted risk estimates can be used to identify people at higher risk, to inform shared decision making between healthcare professionals and service users, or for population level stratification.

### Strengths and limitations of study

Our study has some major strengths, but some important limitations, which include the specific factors related to covid-19 along with others that are similar to those for a range of other widely used clinical risk prediction algorithms developed using the QResearch database.^14^ ^15^ ^16^ Key strengths include the use of a very large validated data source that has been used to develop other risk prediction tools; the wealth of candidate risk predictors; the prospective recording of outcomes and their ascertainment using multiple national level database linkage; lack of selection, recall and respondent biases; and robust statistical analysis. We have used non-linear terms for body mass index and age. We examined interaction terms, which show increased risks at younger ages for adults with type 2 diabetes. We also established a new linkage to the systemic anti-cancer therapy (SACT) database for chemotherapy prescribed and administered in secondary care (which may not be recorded well in general practice software) to circumvent possible missing data for this important variable.

Specific limitations include the occurrence of shielding during the study period and that the study was conducted during the first phase of the UK epidemic. We have accounted for many risk factors for covid-19 mortality, but risks may be conferred by some rare medical conditions or other factors such as occupation that have not yet been observed or are poorly recorded in general practice or hospital data. In particular, the model does not include two important predictors—namely, prevailing infection rate and personal social distancing measures. A lack of comprehensive testing has led to some missing data on covid-19 admissions and/or deaths, which means that development of a valid model for predicting death in people infected with SARS-CoV-2 is not yet possible. We acknowledge that absolute risks are changing during the course of the pandemic, so these should be interpreted with caution. However, we would expect predictors of risk, relative risk measures, and discrimination to be more stable over time, which is consistent with the results from our temporal validation. Although this tool was modelled on the best available data from the first wave of the pandemic, it will be updated as further testing and outcome data accrue, immunity levels change, and (potentially) a vaccine becomes available. Nevertheless, having a risk score available at this stage of the pandemic may be useful to identify people at high risk before a vaccine or treatment is available.

We have reported a validation in each of two time periods using practices from QResearch, but these practices were completely separate from those used to develop the model. We have used this approach previously to develop and validate other widely used prediction models. When these have been further externally validated on completely different clinical databases, by ourselves and others, the results have been very similar.^33^ ^34^ ^35^ Work is already under way to evaluate the models in external datasets across all four nations of the UK and to integrate the algorithms within NHS clinical software systems.

### Policy implication and conclusions

This study presents robust risk prediction models that could be used to stratify risk in populations for public health purposes in the event of a “second wave” of the pandemic and support shared management of risk. We anticipate that the algorithms will be updated regularly as understanding of covid-19 increases, as more data become available, as behaviour in the population changes, or in response to new policy interventions. It is important for patients/carers and clinicians that a common, appropriately developed, evidence based model exists that is consistently implemented and is supported by the academic, clinical, and patient communities. This will then help to ensure consistent policy and clear national communication between policy makers, professionals, employers, and the public.

**What is already known on this topic**

Public policy measures and clinical risk assessment relevant to covid-19 can be aided by rigorously developed and validated risk prediction models
Published risk prediction models for covid-19 are subject to a high risk of bias with optimistic reported performance, raising concern that these models may be unreliable when applied in practice

**What this study adds**

Novel clinical risk prediction models (QCOVID) have been developed and evaluated to identify risks of short term severe outcomes due to covid-19
The risk models have excellent discrimination and are well calibrated; they will be regularly updated as the absolute risks change over time
QCOVID has the potential to support public health policy by enabling shared decision making between clinicians and patients, targeted recruitment for clinical trials, and prioritisation for vaccination

## Web extra

Extra material supplied by authors

Web appendix: Supplementary materials

## Notes

Contributors: JHC, CC, AKC, RK, KDO, PH, and NM led study conceptualisation. All authors contributed to the development of the research question and study design, with development of advanced statistical aspects led by JHC, CC, RK, KDO, and AKC. JHC, AKC, CC, JB, and PJ were involved in data specification, curation, and collection. JHC and AKC developed, checked, or updated clinical code groups. JHC did the statistical analyses, which were checked by CC. JHC developed the software for the web calculator. All authors contributed to the interpretation of the results. AKC and JHC wrote the first draft of the paper. All authors contributed to the critical revision of the manuscript for important intellectual content and approved the final version of the manuscript. The corresponding author attests that all listed authors meet authorship criteria and that no others meeting the criteria have been omitted. JHC is the guarantor.

Ethical approval: The QResearch ethics approval is with East Midlands-Derby Research Ethics Committee (reference 18/EM/0400).

Data sharing: To guarantee the confidentiality of personal and health information, only the authors have had access to the data during the study in accordance with the relevant licence agreements. Access to the QResearch data is according to the information on the QResearch website (www.qresearch.org).

The lead author affirms that the manuscript is an honest, accurate, and transparent account of the study being reported; that no important aspects of the study have been omitted; and that any discrepancies from the study as planned (and, if relevant, registered) have been explained.

Dissemination to participants and related patient and public communities: Patient representatives from the QResearch Advisory Board have advised on dissemination of studies using QResearch data, including the use of lay summaries describing the research and its findings.

Provenance and peer review: Not commissioned; externally peer reviewed.
