# Supplementary material for: Living risk prediction algorithm (QCOVID) for risk of hospital admission and mortality from coronavirus 19 in adults: national derivation and validation cohort study
Source: BMJ. 2020 Oct 21;371:m3731. doi: 10.1136/bmj.m3731 (PMC7574532; doi:10.1136/bmj.m3731)
Supplement: Supplementary file 1 — Web appendix: Supplementary materials [file clia060842.ww1.pdf]

**Supplementary materials**

**Supplementary Box A: Scale of myeloimmunosuppression by chemotherapy drugs/regimens based on the risk of Grade 3/4 febrile neutropenia (CTCv4) or lymphopenia**

| **Group A** | **Group B** | **Group C** |
|---|---|---|
| **<10 %** | **10-50%** | **>50%** |
| • Trastuzumab +/- pertuzumab<br>• Abiraterone<br>• Enzalutamide<br>• Bisphosphonate<br>• Denosumab<br>• Aromatase inhibitors<br>• Fulvestrant<br>• Tamoxifen<br>• Single agent:<br>Atezolizumab<br>Pembrolizumab<br>Ipilimumab<br>Nivolumab<br>• Cetuximab<br>• Panitumumab<br>• Durvalumab<br>• Anagrelide<br>• Taxane – weekly<br>• Cisplatin based regimens<br>• CDK4/6 inhibitors<br>• 5FU single agent<br>• Mitomycin C<br>• Capecitabine single agent<br>• Bevacizumab single agent<br>• Pemetrexed<br>• mTOR inhibitors<br>• Raltitrexed<br>• Methotrexate<br>• PARP inhibitors<br>• Lenvatinib<br>• Sorafenib<br>• Regorafinib<br>• Tyrosine kinase inhibitors (including ALK &/or ROS)<br>• Hydroxycarbamide<br>• Busulfan<br>• Interferon (all formulations) | • Etoposide based regimens<br>• CMF<br>• Irinotecan and Oxaliplatin based regimens<br>• Cabazitaxel<br>• Gemcitabine<br>• Chorambucil<br>• Temozolomide<br>• Daratumumab<br>• Rituximab<br>• Obinutuzumab<br>• Pentostatin<br>• Proteosome inhibitors<br>• IMIDs<br>• PI3Kinase inhibitors<br>• BTK inhibitors<br>• JAK inhibitors<br>• Ventoclax<br>• Trastuzumab-emtansine<br>• Anthracycline based regimens<br>• FEC<br>• MVAC<br>• ABVD<br>• CHOP<br>• BEACOPP<br>• Liposomal doxorubicin<br>• Taxane – 3 weekly<br>• Nab-paclitaxel<br>• Carboplatin based regimens<br>• Ifophosphamide based regimens<br>• Bendamustine<br>• Cladrabine<br>• Topotecan<br>• Cyclophosphamide/Fludarabine combinations<br>• ICE<br>• GDP | • All ALL/AML regimens<br>• BEP<br>• Highly immunosuppressive chemotherapy (e.g. FluDAP, high dose Methotrexate & Cytarabine)<br>• Trifuradine/ Tipracil |

| | | |
|---|---|---|
| | • DHAP<br>• ESHAP<br>• CVAD<br>• Dacarbazine based regimens<br>• Lomustine<br>• Mogalizumab<br>• Brentuximab vedotin<br>• Asparaginase based regimens | |

**Supplementary Table A: Baseline characteristics of the validation cohort for period 1 (24.01.2020 to 30.04.2020)**

| | *Validation cohort total* (%) | *Validation COVID deaths* | *Validation COVID admission* |
|---|---|---|---|
| total | 2173056 | 1722 | 3703 |
| males | 1075788 (49.51) | 978 (56.79) | 2076 (56.06) |
| mean age (SD) | 48.08 (18.69) | 80.40 (11.67) | 70.56 (17.23) |
| 19-29 years | 424125 (19.52) | * | 81 (2.19) |
| 30-39 years | 417590 (19.22) | * | 143 (3.86) |
| 40-49 years | 358695 (16.51) | 22 (1.28) | 269 (7.26) |
| 50-59 years | 358820 (16.51) | 77 (4.47) | 470 (12.69) |
| 60-69 years | 270340 (12.44) | 183 (10.63) | 568 (15.34) |
| 70-79 years | 209557 (9.64) | 390 (22.65) | 759 (20.50) |
| 80-89 years | 106349 (4.89) | 655 (38.04) | 993 (26.82) |
| 90+ years | 27580 (1.27) | 389 (22.59) | 420 (11.34) |
| | | | |
| **Geographical region** | | | |
| East Midlands | 56377 (2.59) | 45 (2.61) | 85 (2.30) |
| East of England | 109558 (5.04) | 78 (4.53) | 173 (4.67) |
| London | 483127 (22.23) | 511 (29.67) | 1164 (31.43) |
| North East | 31768 (1.46) | 26 (1.51) | 78 (2.11) |
| North West | 393451 (18.11) | 363 (21.08) | 718 (19.39) |
| South Central | 280072 (12.89) | 252 (14.63) | 427 (11.53) |
| South East | 239836 (11.04) | 135 (7.84) | 338 (9.13) |
| South West | 319539 (14.70) | 111 (6.45) | 296 (7.99) |
| West Midlands | 171473 (7.89) | 142 (8.25) | 317 (8.56) |
| Yorkshire & Humber | 87855 (4.04) | 59 (3.43) | 107 (2.89) |
| | | | |
| **Ethnicity** | | | |
| White | 1420278 (65.36) | 1169 (67.89) | 2380 (64.27) |
| Indian | 50831 (2.34) | 39 (2.26) | 108 (2.92) |
| Pakistani | 32866 (1.51) | 24 (1.39) | 75 (2.03) |
| Bangladeshi | 23424 (1.08) | 22 (1.28) | 50 (1.35) |
| Other Asian | 34412 (1.58) | 21 (1.22) | 72 (1.94) |
| Caribbean | 24135 (1.11) | 68 (3.95) | 138 (3.73) |
| Black African | 47933 (2.21) | 37 (2.15) | 135 (3.65) |
| Chinese | 23885 (1.10) | * | 18 (0.49) |
| Other ethnic group | 81009 (3.73) | 25 (1.45) | 135 (3.65) |
| Ethnicity not recorded | 434283 (19.98) | 308 (17.89) | 592 (15.99) |
| | | | |
| **Townsend quintile** | | | |
| 1 (most affluent) | 446359 (20.54) | 312 (18.12) | 697 (18.82) |
| 2 | 428735 (19.73) | 316 (18.35) | 630 (17.01) |
| 3 | 439846 (20.24) | 373 (21.66) | 736 (19.88) |
| 4 | 436574 (20.09) | 318 (18.47) | 770 (20.79) |

| | | | |
|---|---|---|---|
| 5 (most deprived) | 409917 (18.86) | 400 (23.23) | 861 (23.25) |
| Townsend not recorded | 11625 (0.53) | * | * |
| | | | |
| **accommodation** | | | |
| neither | 2155199 (99.18) | 1190 (69.11) | 3316 (89.55) |
| Care or residential home | 14057 (0.65) | 529 (30.72) | 373 (10.07) |
| Homeless | 3800 (0.17) | * | * |
| **Body mass index ($kg/m^2$)** | | | |
| BMI < 18.5 | 59376 (2.73) | 86 (4.99) | 113 (3.05) |
| BMI 18.5-24.99 | 711186 (32.73) | 575 (33.39) | 955 (25.79) |
| BMI 25-29.99 | 596942 (27.47) | 498 (28.92) | 1183 (31.95) |
| BMI 30-34.99 | 278830 (12.83) | 256 (14.87) | 704 (19.01) |
| BMI 35+ | 160345 (7.38) | 171 (9.93) | 530 (14.31) |
| BMI not recorded | 366377 (16.86) | 136 (7.90) | 218 (5.89) |
| | | | |
| **smoking status** | | | |
| non smoker | 1242933 (57.20) | 895 (51.97) | 2087 (56.36) |
| ex smoker | 460999 (21.21) | 671 (38.97) | 1285 (34.70) |
| light smoker | 290738 (13.38) | 91 (5.28) | 231 (6.24) |
| moderate smoker | 57297 (2.64) | 14 (0.81) | 27 (0.73) |
| heavy smoker | 26332 (1.21) | 10 (0.58) | 26 (0.70) |
| Smoking not recorded | 94757 (4.36) | 41 (2.38) | 47 (1.27) |
| | | | |
| no CKD | 2087614 (96.07) | 1200 (69.69) | 2852 (77.02) |
| CKD3 | 76600 (3.52) | 431 (25.03) | 667 (18.01) |
| CKD4 | 4648 (0.21) | 40 (2.32) | 73 (1.97) |
| CKD5 only | 2527 (0.12) | 35 (2.03) | 78 (2.11) |
| CKD5 with dialysis | 585 (0.03) | 12 (0.70) | 16 (0.43) |
| CKD5 with transplant | 1082 (0.05) | * | 17 (0.46) |
| | | | |
| no learning disability | 2137759 (98.38) | 1645 (95.53) | 3572 (96.46) |
| Learning disability | 34257 (1.58) | 75 (4.36) | 125 (3.38) |
| Down's Syndrome | 1040 (0.05) | * | * |
| | | | |
| No chemotherapy in last 12 | 2164341 (99.60) | 1687 (97.97) | 3603 (97.30) |
| Chemotherapy group A | 3343 (0.15) | 7 (0.41) | 24 (0.65) |
| Chemotherapy group B | 5032 (0.23) | 24 (1.39) | 71 (1.92) |
| Chemotherapy group C | 340 (0.02) | * | * |
| | | | |
| **Cancer and immunosuppression** | | | |
| Blood cancer | 10359 (0.48) | 49 (2.85) | 89 (2.40) |
| Bone marrow or stem cell transplant in last 6 months | 73 (0.00) | * | * |
| Respiratory cancer | 4549 (0.21) | 21 (1.22) | 46 (1.24) |

| | | | |
|---|---|---|---|
| Radiotherapy in last 6 months | 4346 (0.20) | 15 (0.87) | 39 (1.05) |
| Solid organ transplant | 1147 (0.05) | * | 15 (0.41) |
| Prescribed immunosuppressants by GP | 2814 (0.13) | 10 (0.58) | 13 (0.35) |
| Prescribed leukotriene or LABA | 45905 (2.11) | 155 (9.00) | 311 (8.40) |
| prescribed regular prednisolone | 11617 (0.53) | 68 (3.95) | 149 (4.02) |
| Sickle cell disease | 717 (0.03) | * | * |
| | | | |
| **Other co-morbidities** | | | |
| Type 1 diabetes | 10337 (0.48) | 13 (0.75) | 59 (1.59) |
| Type 2 diabetes | 137092 (6.31) | 518 (30.08) | 1048 (28.30) |
| | | | |
| COPD | 51026 (2.35) | 246 (14.29) | 400 (10.80) |
| asthma | 299632 (13.79) | 231 (13.41) | 618 (16.69) |
| Rare pulmonary diseases | 11748 (0.54) | 55 (3.19) | 97 (2.62) |
| Pulmonary hypertension or pulmonary fibrosis | 1891 (0.09) | 19 (1.10) | 26 (0.70) |
| | | | |
| Coronary heart disease | 77035 (3.55) | 368 (21.37) | 626 (16.91) |
| Stroke | 47513 (2.19) | 359 (20.85) | 475 (12.83) |
| Atrial Fibrillation | 52764 (2.43) | 294 (17.07) | 515 (13.91) |
| Congestive cardiac failure | 25255 (1.16) | 224 (13.01) | 349 (9.42) |
| Venous thromboembolism | 38962 (1.79) | 157 (9.12) | 290 (7.83) |
| Peripheral vascular disease | 16463 (0.76) | 101 (5.87) | 178 (4.81) |
| Congenital heart disease | 11344 (0.52) | 20 (1.16) | 37 (1.00) |
| | | | |
| Dementia | 21984 (1.01) | 603 (35.02) | 521 (14.07) |
| Parkinson's disease | 5736 (0.26) | 68 (3.95) | 74 (2.00) |
| Epilepsy | 29031 (1.34) | 53 (3.08) | 131 (3.54) |
| Rare neurological conditions | 6804 (0.31) | 26 (1.51) | 37 (1.00) |
| Cerebral palsy | 2433 (0.11) | * | * |
| Severe mental illness | 246668 (11.35) | 333 (19.34) | 681 (18.39) |
| Osteoporotic fracture | 87595 (4.03) | 301 (17.48) | 413 (11.15) |
| Rheumatoid arthritis or SLE | 21391 (0.98) | 51 (2.96) | 107 (2.89) |
| Cirrhosis of the liver | 4442 (0.20) | * | 34 (0.92) |
| On the shielded list | 88170 (4.06) | 364 (21.14) | 817 (22.06) |

* represents values which have been suppressed due to small numbers < 15

**Supplementary Table B: Baseline characteristics of the validation cohort for period 2 (01.05.2020 to 30.06.2020)**

| category | COVID related deaths | COVID related admissions |
|---|---|---|
| total | 621 | 1002 |
| males | 324 (52.17) | 478 (47.70) |
| mean age (SD) | 82.29 (11.29) | 68.37 (20.92) |
| 19-29 years | * | 57 (5.69) |
| 30-39 years | * | 89 (8.88) |
| 40-49 years | * | 65 (6.49) |
| 50-59 years | 24 (3.86) | 110 (10.98) |
| 60-69 years | 45 (7.25) | 104 (10.38) |
| 70-79 years | 118 (19.00) | 163 (16.27) |
| 80+ years | 250 (40.26) | 281 (28.04) |
| 90+ years | 176 (28.34) | 133 (13.27) |
| | | |
| **Geographical region** | | |
| East Midlands | 39 (6.28) | 49 (4.89) |
| East of England | 21 (3.38) | 53 (5.29) |
| London | 81 (13.04) | 126 (12.57) |
| North East | * | * |
| North West | 175 (28.18) | 294 (29.34) |
| South Central | 51 (8.21) | 95 (9.48) |
| South East | 77 (12.40) | 130 (12.97) |
| South West | 81 (13.04) | 117 (11.68) |
| West Midlands | 73 (11.76) | 91 (9.08) |
| Yorkshire & Humber | 22 (3.54) | 41 (4.09) |
| | | |
| **Ethnicity** | | |
| White | 459 (73.91) | 700 (69.86) |
| Indian | * | * |
| Pakistani | * | 19 (1.90) |
| Bangladeshi | * | * |
| Other Asian | * | * |
| Caribbean | * | * |
| Black african | * | 15 (1.50) |
| Chinese | * | * |
| Other ethnic group | * | 19 (1.90) |
| Ethnicity not record | 131 (21.10) | 206 (20.56) |
| | | |
| **Townsend quintile** | | |
| 1 (most affluent) | 168 (27.05) | 223 (22.26) |
| 2 | 112 (18.04) | 187 (18.66) |
| 3 | 148 (23.83) | 217 (21.66) |

| | | |
|---|---|---|
| 4 | 110 (17.71) | 219 (21.86) |
| 5 (most deprived) | 80 (12.88) | 154 (15.37) |
| Townsend not recorded | * | * |
| | | |
| **accommodation** | | |
| neither | 405 (65.22) | 855 (85.33) |
| carehome | 215 (34.62) | 146 (14.57) |
| Homeless | * | * |
| | | |
| BMI < 18 | 36 (5.80) | 31 (3.09) |
| BMI 18-24.99 | 230 (37.04) | 303 (30.24) |
| BMI 25-29.99 | 177 (28.50) | 302 (30.14) |
| BMI 30-34.99 | 66 (10.63) | 170 (16.97) |
| BMI 35+ | 58 (9.34) | 124 (12.38) |
| BMI not recorded | 54 (8.70) | 72 (7.19) |
| | | |
| smoking status | | |
| non smoker | 321 (51.69) | 558 (55.69) |
| ex smoker | 234 (37.68) | 317 (31.64) |
| light smoker | 35 (5.64) | 83 (8.28) |
| moderate smoker | * | 15 (1.50) |
| heavy smoker | * | * |
| Smoking not recorded | 23 (3.70) | 19 (1.90) |
| | | |
| no CKD | 423 (68.12) | 780 (77.84) |
| CKD3 | 178 (28.66) | 180 (17.96) |
| CKD4 | * | 18 (1.80) |
| CKD5 only | * | 15 (1.50) |
| CKD5 with dialysis | * | * |
| CKD5 with transplant | * | * |
| | | |
| no learning disabili | 600 (96.62) | 970 (96.81) |
| Learning disability | 20 (3.22) | 31 (3.09) |
| Downs | | |
| | | |
| No chemo in last 12 | 601 (96.78) | 984 (98.20) |
| Chemo group A | * | * |
| chemo group B | * | * |
| chemo group C | | * |
| | | |
| **Cancer and immunosuppression** | | |
| Blood cancer | * | 15 (1.50) |
| Bone marrow or stem cell transplant in last 6 months | * | * |
| Respiratory cancer | * | * |

| | | |
|---|---|---|
| Radiotherapy in last 6 months | * | * |
| Solid organ transplant | * | * |
| Prescribed immunosuppressant medication by GP | * | * |
| Prescribed leukotriene or LABA | 54 (8.70) | 59 (5.89) |
| prescribed regular prednisolone | 24 (3.86) | 33 (3.29) |
| Sickle cell disease | * | * |
| | | |
| **Other co-morbidities** | | |
| Type 1 diabetes | * | * |
| Type 2 diabetes | 172 (27.70) | 233 (23.25) |
| | | |
| COPD | 74 (11.92) | 94 (9.38) |
| asthma | 83 (13.37) | 167 (16.67) |
| Rare pulmonary diseases | 17 (2.74) | 19 (1.90) |
| Pulmonary hypertension or pulmonary fibrosis | * | * |
| | | |
| Coronary heart disease | 145 (23.35) | 173 (17.27) |
| Stroke | 126 (20.29) | 160 (15.97) |
| Atrial Fibrillation | 132 (21.26) | 157 (15.67) |
| Congestive cardiac failure | 65 (10.47) | 89 (8.88) |
| Venous thromboembolism | 53 (8.53) | 80 (7.98) |
| Peripheral vascular disease | 36 (5.80) | 43 (4.29) |
| Congenital heart disease | * | * |
| | | |
| Dementia | 255 (41.06) | 174 (17.37) |
| Parkinson's disease | 34 (5.48) | 26 (2.59) |
| Epilepsy | 26 (4.19) | 35 (3.49) |
| Rare neurological conditions | * | * |
| Cerebral palsy | * | * |
| Severe mental illness | 110 (17.71) | 195 (19.46) |
| Osteoporotic fracture | 123 (19.81) | 119 (11.88) |
| Rheumatoid arthritis or SLE | 22 (3.54) | 30 (2.99) |
| Cirrhosis of the liver | * | 18 (1.80) |

**Supplementary Table C: Performance of the risk models to predict risk of COVID-19 death in the validation cohort by subgroup using Harrell's C statistic (95% CI) in study period 1 (24.01.2020 to 30.04.2020) and period 1 (01.05.2020 to 30.06.2020)**

| | **period 1** | **period 1** | **period 2** | **period 2** |
|---|---|---|---|---|
| | **death** | **death** | **death** | **death** |
| groupcat | **females** | **males** | **females** | **males** |
| overall | .933 (.923 to .944) | .928 (.919 to .938) | .952 (.938 to .965) | .933 (.918 to .949) |
| <50 years | * | * | * | * |
| 50-59 years | .618 (.519 to .717) | .678 (.612 to .744) | .517 (.516 to .517) | .717 (.601 to .833) |
| 60-69 years | .77 (.712 to .828) | .831 (.79 to .872) | .794 (.678 to .91) | .753 (.665 to .84) |
| 70-79 years | .866 (.832 to .9) | .812 (.782 to .841) | .845 (.78 to .91) | .845 (.793 to .897) |
| 80+ years | .821 (.803 to .838) | .814 (.796 to .833) | .817 (.79 to .843) | .801 (.771 to .831) |
| | | | | |
| East Midlands | .952 (.895 to 1.01) | .927 (.865 to .989) | .943 (.89 to .996) | .927 (.86 to .994) |
| East of England | .925 (.874 to .976) | .904 (.849 to .959) | .905 (.799 to 1.01) | .96 (.932 to .989) |
| London | .937 (.917 to .958) | .912 (.893 to .932) | .967 (.936 to .997) | .936 (.894 to .978) |
| North East | .971 (.947 to .994) | .957 (.937 to .976) | .975 (.973 to .977) | * |
| North West | .929 (.905 to .953) | .942 (.924 to .959) | .945 (.917 to .973) | .925 (.89 to .961) |
| South Central | .946 (.922 to .971) | .947 (.928 to .966) | .94 (.9 to .981) | .88 (.81 to .949) |
| South East | .918 (.879 to .958) | .918 (.881 to .955) | .94 (.893 to .986) | .955 (.93 to .981) |
| South West | .952 (.918 to .985) | .931 (.898 to .965) | .961 (.925 to .996) | .937 (.892 to .982) |
| West Midlands | .907 (.864 to .949) | .923 (.887 to .958) | .958 (.924 to .992) | .924 (.872 to .975) |
| Yorkshire & Humber | .92 (.842 to .998) | .98 (.972 to .988) | .993 (.989 to .996) | .969 (.948 to .99) |
| | | | | |
| White | .932 (.921 to .944) | .93 (.92 to .94) | .953 (.94 to .967) | .936 (.92 to .952) |
| Indian | .915 (.842 to .987) | .886 (.802 to .97) | .987 (.975 to .999) | .864 (.666 to 1.06) |
| Pakistani | .915 (.805 to 1.03) | .933 (.869 to .996) | * | .849 (.654 to 1.04) |
| Bangladeshi | .954 (.923 to .986) | .916 (.845 to .987) | .72 (.333 to 1.11) | .991 (.98 to 1) |
| Other Asian | .862 (.701 to 1.02) | .91 (.835 to .985) | * | .948 (.945 to .951) |
| Caribbean | .96 (.941 to .978) | .938 (.903 to .973) | .919 (.834 to 1) | .97 (.94 to .999) |
| Black african | .877 (.774 to .981) | .915 (.851 to .978) | .905 (.901 to .908) | .947 (.89 to 1) |
| Chinese | .981 (.966 to .996) | .976 (.955 to .998) | * | * |
| Other ethnic group | .981 (.97 to .992) | .884 (.796 to .972) | .858 (.63 to 1.09) | .867 (.709 to 1.02) |
| | | | | |
| 1 (most affluent) | .941 (.919 to .964) | .922 (.898 to .945) | .925 (.89 to .96) | .936 (.907 to .965) |
| 2 | .947 (.926 to .969) | .922 (.898 to .946) | .963 (.938 to .987) | .944 (.915 to .973) |
| 3 | .918 (.892 to .944) | .927 (.907 to .947) | .964 (.943 to .985) | .912 (.869 to .955) |
| 4 | .936 (.911 to .961) | .924 (.901 to .946) | .957 (.926 to .988) | .939 (.905 to .973) |
| 5 (most deprived) | .927 (.904 to .951) | .944 (.927 to .961) | .96 (.93 to .991) | .932 (.889 to .974) |

* could not be evaluated due to small number of events

**Supplementary Table D: Performance of the risk models to predict risk of COVID-19 hospital admission in the validation cohort by subgroup using Harrell's C statistic (95% CI) in study period 1 (24.01.2020 to 30.04.2020) and period 2 (01.05.2020 to 30.06.2020)**

| | **period 1** | **period 1** | **period 2** | **period 2** |
|---|---|---|---|---|
| | **hospital** | **hospital** | **hospital** | **hospital** |
| groupcat | **females** | **males** | **females** | **males** |
| overall | .847 (.836 to .857) | .86 (.852 to .868) | .776 (.753 to .8) | .833 (.812 to .853) |
| <50 years | .693 (.659 to .726) | .744 (.713 to .775) | .689 (.601 to .777) | .66 (.569 to .751) |
| 50-59 years | .744 (.708 to .779) | .695 (.664 to .727) | .667 (.597 to .738) | .686 (.621 to .75) |
| 60-69 years | .73 (.696 to .765) | .771 (.745 to .797) | .672 (.575 to .768) | .746 (.69 to .803) |
| 70-79 years | .801 (.773 to .828) | .764 (.742 to .786) | .792 (.743 to .841) | .769 (.721 to .817) |
| 80+ years | .712 (.693 to .73) | .717 (.698 to .735) | .715 (.685 to .746) | .714 (.677 to .751) |
| | | | | |
| East Midlands | .867 (.812 to .922) | .9 (.861 to .939) | .817 (.73 to .903) | .858 (.782 to .935) |
| East of England | .824 (.773 to .876) | .838 (.8 to .875) | .804 (.704 to .904) | .874 (.798 to .95) |
| London | .854 (.835 to .873) | .868 (.855 to .882) | .696 (.625 to .767) | .869 (.814 to .924) |
| North East | .776 (.69 to .862) | .848 (.792 to .905) | .848 (.687 to 1.01) | .948 (.918 to .978) |
| North West | .859 (.837 to .88) | .86 (.841 to .88) | .812 (.77 to .853) | .82 (.781 to .858) |
| South Central | .825 (.793 to .856) | .867 (.845 to .889) | .753 (.679 to .827) | .812 (.742 to .882) |
| South East | .864 (.834 to .895) | .836 (.805 to .867) | .771 (.704 to .838) | .849 (.788 to .909) |
| South West | .812 (.777 to .847) | .825 (.792 to .858) | .751 (.676 to .827) | .827 (.772 to .882) |
| West Midlands | .843 (.808 to .877) | .833 (.801 to .865) | .789 (.71 to .867) | .808 (.736 to .88) |
| Yorkshire & Humber | .867 (.812 to .922) | .941 (.922 to .96) | .79 (.673 to .908) | .83 (.746 to .914) |
| | | | | |
| White | .852 (.84 to .863) | .863 (.854 to .872) | .786 (.761 to .81) | .848 (.827 to .869) |
| Indian | .822 (.755 to .889) | .796 (.743 to .849) | .749 (.606 to .892) | .836 (.712 to .96) |
| Pakistani | .735 (.636 to .834) | .8 (.742 to .858) | .836 (.635 to 1.04) | .693 (.557 to .83) |
| Bangladeshi | .696 (.575 to .818) | .843 (.753 to .934) | .79 (.581 to 1) | .783 (.511 to 1.05) |
| Other Asian | .748 (.669 to .827) | .851 (.803 to .9) | .584 (.348 to .819) | .53 (.332 to .728) |
| Caribbean | .865 (.825 to .905) | .864 (.825 to .902) | .845 (.669 to 1.02) | .933 (.877 to .989) |
| Black african | .855 (.805 to .905) | .84 (.797 to .883) | .566 (.411 to .721) | .688 (.537 to .84) |

| | | | | |
|---|---|---|---|---|
| Chinese | .87 (.739 to 1) | .894 (.804 to .985) | * | .983 (.975 to .992) |
| Other ethnic group | .796 (.74 to .852) | .84 (.798 to .883) | .546 (.385 to .706) | .703 (.539 to .867) |
| | | | | |
| 1 (most affluent) | .834 (.81 to .859) | .826 (.805 to .847) | .821 (.774 to .868) | .878 (.844 to .913) |
| 2 | .855 (.832 to .879) | .855 (.834 to .875) | .753 (.695 to .811) | .827 (.777 to .876) |
| 3 | .84 (.817 to .863) | .859 (.841 to .877) | .785 (.734 to .836) | .802 (.754 to .85) |
| 4 | .838 (.814 to .861) | .867 (.85 to .884) | .779 (.731 to .827) | .835 (.789 to .88) |
| 5 (most deprived) | .858 (.837 to .878) | .883 (.867 to .9) | .728 (.665 to .791) | .818 (.766 to .871) |

*could not be evaluated due to small number of events

**Supplementary Table E: Performance of the risk models to predict risk of COVID-19 death in the validation cohort by subgroup (D statistic and $R^2$ explained variation) in study period 1 (24.01.2020 to 30.04.2020) and period 2 (01.05.2020 to 30.06.2020)**

| | | **period 1** | **period1** | **period 2** | **period 2** |
|---|---|---|---|---|---|
| | | **death** | **death** | **death** | **death** |
| statistic | group | **females** | **males** | **females** | **males** |
| | | | | | |
| R2 | overall | 74 (72.7 to 75.3) | 73.1 (71.9 to 74.3) | 75.4 (73.5 to 77.4) | 73.6 (71.6 to 75.6) |
| D statistic | overall | 3.46 (3.34 to 3.57) | 3.37 (3.27 to 3.47) | 3.59 (3.4 to 3.77) | 3.42 (3.24 to 3.59) |
| | | | | | |
| R2 | <50 years | 67.9 (53.3 to 82.5) | 54.2 (37.7 to 70.7) | * | 49.2 (3.38 to 95) |
| D statistic | <50 years | 2.98 (1.98 to 3.97) | 2.23 (1.49 to 2.97) | * | 2.02 (.172 to 3.87) |
| R2 | 50-59 years | 53.2 (37.2 to 69.2) | 52.4 (42.6 to 62.1) | * | 50.6 (33.1 to 68) |
| D statistic | 50-59 years | 2.18 (1.48 to 2.88) | 2.15 (1.73 to 2.56) | 1.5 (.101 to 2.9) | 2.07 (1.35 to 2.79) |
| R2 | 60-69 years | 50.6 (41.6 to 59.6) | 57 (50.8 to 63.2) | 51.6 (33.6 to 69.6) | 42.3 (25.8 to 58.9) |
| D statistic | 60-69 years | 2.07 (1.7 to 2.44) | 2.36 (2.06 to 2.66) | 2.11 (1.35 to 2.87) | 1.75 (1.16 to 2.35) |
| R2 | 70-79 years | 62.8 (57.9 to 67.8) | 53.2 (48.7 to 57.7) | 62.6 (54.2 to 71) | 56.7 (48.9 to 64.6) |
| D statistic | 70-79 years | 2.66 (2.38 to 2.94) | 2.18 (1.99 to 2.38) | 2.65 (2.18 to 3.13) | 2.34 (1.97 to 2.72) |
| R2 | 80+ years | 47.7 (44.2 to 51.3) | 47.6 (44.1 to 51.1) | 46.6 (41.1 to 52) | 44.7 (38.6 to 50.7) |
| D statistic | 80+ years | 1.96 (1.82 to 2.09) | 1.95 (1.81 to 2.09) | 1.91 (1.7 to 2.12) | 1.84 (1.61 to 2.06) |
| | | | | | |
| R2 | White | 73.5 (72 to 74.9) | 73.4 (72.1 to 74.6) | 75.3 (73.3 to 77.2) | 74.1 (72.1 to 76.2) |
| D statistic | White | 3.4 (3.28 to 3.53) | 3.4 (3.28 to 3.51) | 3.57 (3.38 to 3.76) | 3.46 (3.28 to 3.65) |
| R2 | Indian | 73.4 (65.1 to 81.7) | 70.6 (61 to 80.2) | 76.8 (51 to 103) | 68.8 (47.9 to 89.6) |
| D statistic | Indian | 3.4 (2.68 to 4.12) | 3.17 (2.45 to 3.9) | 3.75 (1.23 to 6.28) | 3.05 (1.58 to 4.53) |
| R2 | Pakistani | 72.1 (56.2 to 88) | 70.7 (60.8 to 80.6) | * | 66.6 (41.3 to 92) |
| D statistic | Pakistani | 3.3 (2 to 4.6) | 3.18 (2.42 to 3.94) | * | 2.91 (1.24 to 4.58) |
| R2 | Bangladeshi | 70.4 (49.6 to 91.3) | 71.1 (61.3 to 80.8) | 68.7 (36.1 to 101) | 75.9 (43.4 to 108) |
| D statistic | Bangladeshi | 3.16 (1.6 to 4.73) | 3.21 (2.45 to 3.97) | 3.03 (.735 to 5.32) | 3.67 (.477 to 6.86) |
| R2 | Other Asian | 78.4 (68.4 to 88.5) | 68.2 (55.8 to 80.6) | * | 62 (4.65 to 119) |

| | | | | | |
|---|---|---|---|---|---|
| D statistic | Other Asian | 3.9 (2.74 to 5.07) | 3 (2.14 to 3.85) | * | 2.64 (-.421 to 5.71) |
| R2 | Caribbean | 74.5 (68.2 to 80.8) | 69.6 (62.5 to 76.7) | 62 (21.1 to 103) | 75.4 (57.2 to 93.5) |
| D statistic | Caribbean | 3.5 (2.92 to 4.08) | 3.1 (2.58 to 3.62) | 2.66 (.389 to 4.94) | 3.59 (1.82 to 5.36) |
| R2 | Black african | 77.4 (68.6 to 86.1) | 69.4 (60.6 to 78.2) | * | 70.7 (48.4 to 92.9) |
| D statistic | Black african | 3.78 (2.84 to 4.73) | 3.08 (2.44 to 3.72) | * | 3.2 (1.53 to 4.88) |
| R2 | Chinese | 84.2 (71.3 to 97.2) | 80.2 (69.1 to 91.4) | * | * |
| D statistic | Chinese | 4.73 (2.42 to 7.04) | 4.13 (2.68 to 5.58) | * | * |
| R2 | Other ethnic group | 78 (68.8 to 87.2) | 71.5 (62.3 to 80.8) | 74 (53.4 to 94.6) | 66.3 (43.5 to 89) |
| D statistic | Other ethnic group | 3.86 (2.83 to 4.89) | 3.25 (2.51 to 3.98) | 3.47 (1.64 to 5.29) | 2.89 (1.45 to 4.33) |
| | | | | | |
| R2 | East Midlands | 75.4 (67.6 to 83.2) | 74.9 (68 to 81.7) | 72.3 (63.7 to 80.9) | 71.2 (61.4 to 80.9) |
| D statistic | East Midlands | 3.58 (2.83 to 4.34) | 3.53 (2.89 to 4.18) | 3.31 (2.6 to 4.01) | 3.21 (2.45 to 3.98) |
| R2 | East of England | 73.4 (67.3 to 79.4) | 70.2 (63.4 to 77) | 71.5 (58.4 to 84.5) | 71.5 (59.1 to 83.9) |
| D statistic | East of England | 3.4 (2.87 to 3.92) | 3.14 (2.63 to 3.65) | 3.24 (2.21 to 4.27) | 3.24 (2.26 to 4.22) |
| R2 | London | 75.8 (73.5 to 78.1) | 72.5 (70.3 to 74.6) | 76.7 (71.5 to 81.9) | 72.7 (67.1 to 78.4) |
| D statistic | London | 3.62 (3.39 to 3.85) | 3.32 (3.14 to 3.5) | 3.71 (3.18 to 4.25) | 3.34 (2.87 to 3.82) |
| R2 | North East | 72.4 (59.3 to 85.6) | 69.1 (58.2 to 80.1) | 69.8 (25.8 to 114) | * |
| D statistic | North East | 3.32 (2.22 to 4.41) | 3.06 (2.28 to 3.85) | * | * |
| R2 | North West | 73.3 (70.5 to 76.2) | 74.4 (71.8 to 76.9) | 75.7 (72.2 to 79.1) | 75.3 (71.8 to 78.9) |
| D statistic | North West | 3.4 (3.15 to 3.64) | 3.48 (3.26 to 3.71) | 3.61 (3.27 to 3.95) | 3.58 (3.23 to 3.92) |
| R2 | South Central | 75.7 (72.7 to 78.7) | 73.5 (70.3 to 76.7) | 70.8 (63 to 78.6) | 69.5 (60 to 78.9) |
| D statistic | South Central | 3.62 (3.32 to 3.91) | 3.41 (3.13 to 3.69) | 3.19 (2.58 to 3.79) | 3.09 (2.4 to 3.78) |
| R2 | South East | 70.4 (65.1 to 75.8) | 72 (67.5 to 76.6) | 74.2 (67.1 to 81.3) | 75.7 (71.2 to 80.3) |
| D statistic | South East | 3.16 (2.75 to 3.57) | 3.28 (2.91 to 3.66) | 3.47 (2.83 to 4.11) | 3.62 (3.17 to 4.07) |
| R2 | South West | 74 (68.9 to 79.1) | 72.5 (67.7 to 77.4) | 77.8 (73.4 to 82.2) | 74.3 (68.6 to 80.1) |
| D statistic | South West | 3.45 (3 to 3.91) | 3.33 (2.92 to 3.73) | 3.83 (3.34 to 4.32) | 3.48 (2.96 to 4.01) |
| R2 | West Midlands | 71.5 (66.3 to 76.6) | 72.2 (67.8 to 76.5) | 74.7 (68.6 to 80.7) | 72.2 (66 to 78.4) |

| | | | | | |
|---|---|---|---|---|---|
| D statistic | West Midlands | 3.24 (2.83 to 3.65) | 3.29 (2.94 to 3.65) | 3.52 (2.95 to 4.08) | 3.3 (2.79 to 3.81) |
| R2 | Yorkshire & Humber | 75.4 (67.6 to 83.1) | 79.2 (74.9 to 83.6) | 80.1 (71.4 to 88.9) | 73.9 (63.8 to 84) |
| D statistic | Yorkshire & Humber | 3.58 (2.83 to 4.33) | 4 (3.47 to 4.53) | 4.11 (2.98 to 5.24) | 3.44 (2.54 to 4.34) |
| | | | | | |
| R2 | Quintile 1 | 73.8 (70.6 to 76.9) | 73.3 (70.5 to 76) | 74.1 (70.2 to 78) | 73.7 (69.8 to 77.6) |
| D statistic | Quintile1 | 3.43 (3.16 to 3.71) | 3.39 (3.15 to 3.63) | 3.46 (3.11 to 3.81) | 3.43 (3.08 to 3.77) |
| R2 | 2 | 75.5 (72.7 to 78.2) | 73.1 (70.3 to 75.9) | 75.6 (70.9 to 80.3) | 74.3 (69.9 to 78.7) |
| D statistic | 2 | 3.59 (3.32 to 3.86) | 3.37 (3.13 to 3.62) | 3.6 (3.14 to 4.06) | 3.48 (3.08 to 3.88) |
| R2 | 3 | 71.1 (67.9 to 74.3) | 72.2 (69.5 to 74.9) | 75.8 (72.1 to 79.4) | 73.7 (69.3 to 78) |
| D statistic | 3 | 3.21 (2.96 to 3.46) | 3.3 (3.08 to 3.52) | 3.62 (3.26 to 3.98) | 3.43 (3.04 to 3.81) |
| R2 | 4 | 73.4 (70.1 to 76.6) | 72.2 (69.4 to 75) | 76.7 (72.3 to 81) | 74.5 (70 to 79) |
| D statistic | 4 | 3.4 (3.12 to 3.68) | 3.3 (3.07 to 3.53) | 3.71 (3.26 to 4.16) | 3.5 (3.08 to 3.92) |
| R2 | Quintile 5 | 76.1 (73.7 to 78.5) | 74.7 (72.4 to 77) | 76.2 (70.8 to 81.5) | 72.4 (66.6 to 78.2) |
| D statistic | Quintile 5 | 3.65 (3.41 to 3.89) | 3.52 (3.3 to 3.73) | 3.66 (3.12 to 4.2) | 3.32 (2.84 to 3.8) |

Note pre-specified age-bands 19-39 years and 40-49 years were combined for these analyses due to small numbers of events

**Supplementary Table F: Performance of the risk models to predict risk of COVID-19 hospital admission in the validation cohort by subgroup (D and $R^2$ explained variation) in study period 1 (24.01.2020 to 30.04.2020) and period 2 (01.05.2020 to 30.06.2020)**

| | | **period 1** | **period 1** | **period 2** | **period 2** |
|---|---|---|---|---|---|
| | | **hospital** | **hospital** | **hospital** | **hospital** |
| statistic | group | **females** | **males** | **females** | **males** |
| | | | | | |
| R2 | overall | 57.1 (55.5 to 58.8) | 58.1 (56.7 to 59.5) | 45.4 (41.7 to 49.1) | 55.4 (52.2 to 58.5) |
| D statistic | overall | 2.36 (2.28 to 2.44) | 2.41 (2.34 to 2.48) | 1.87 (1.73 to 2) | 2.28 (2.14 to 2.42) |
| | | | | | |
| R2 | <50 years | 35.8 (29.7 to 42) | 47.8 (42.8 to 52.9) | 30.2 (13.1 to 47.3) | 26.8 (8.1 to 45.5) |
| D statistic | <50 years | 1.53 (1.32 to 1.73) | 1.96 (1.76 to 2.16) | 1.35 (.799 to 1.89) | 1.24 (.647 to 1.83) |
| R2 | 50-59 years | 42.1 (35.4 to 48.7) | 33.6 (28 to 39.2) | 22 (7.9 to 36.1) | 29.5 (16.3 to 42.7) |
| D statistic | 50-59 years | 1.74 (1.51 to 1.98) | 1.46 (1.27 to 1.64) | 1.09 (.64 to 1.53) | 1.32 (.903 to 1.74) |
| R2 | 60-69 years | 41.3 (35.3 to 47.4) | 40.2 (35.3 to 45) | 27.5 (8.98 to 45.9) | 36.4 (25.5 to 47.3) |
| D statistic | 60-69 years | 1.72 (1.5 to 1.93) | 1.68 (1.51 to 1.85) | 1.26 (.675 to 1.84) | 1.55 (1.18 to 1.91) |
| R2 | 70-79 years | 47.8 (43.1 to 52.5) | 38.2 (33.8 to 42.5) | 39.9 (28.7 to 51) | 40.7 (31.5 to 49.9) |
| D statistic | 70-79 years | 1.96 (1.77 to 2.14) | 1.61 (1.46 to 1.76) | 1.67 (1.28 to 2.05) | 1.69 (1.37 to 2.02) |
| R2 | 80+ years | 26.2 (22.4 to 29.9) | 26.6 (22.9 to 30.3) | 26 (19.4 to 32.6) | 26.8 (19.3 to 34.3) |
| D statistic | 80+ years | 1.22 (1.1 to 1.34) | 1.23 (1.11 to 1.35) | 1.21 (1.01 to 1.42) | 1.24 (1 to 1.48) |
| | | | | | |
| R2 | White | 57.3 (55.5 to 59.2) | 58.6 (57.1 to 60.2) | 47.4 (43.6 to 51.2) | 57.6 (54.2 to 61) |
| D statistic | White | 2.37 (2.28 to 2.46) | 2.44 (2.36 to 2.51) | 1.94 (1.79 to 2.09) | 2.39 (2.22 to 2.55) |
| R2 | Indian | 57.3 (47.2 to 67.3) | 50.1 (40.8 to 59.4) | 37.1 (.609 to 73.7) | 52 (23.5 to 80.4) |
| D statistic | Indian | 2.37 (1.88 to 2.86) | 2.05 (1.67 to 2.43) | 1.58 (.349 to 2.81) | 2.14 (.919 to 3.37) |
| R2 | Pakistani | 39.1 (21.8 to 56.3) | 47.5 (36.1 to 58.8) | 57.8 (33.6 to 82) | 36.7 (5.46 to 68) |
| D statistic | Pakistani | 1.64 (1.05 to 2.23) | 1.95 (1.5 to 2.39) | 2.4 (1.22 to 3.58) | 1.57 (.507 to 2.63) |
| R2 | Bangladeshi | 37.5 (17.8 to 57.2) | 57.5 (45.5 to 69.5) | * | 55.4 (21.6 to 89.1) |
| D statistic | Bangladeshi | 1.59 (.918 to 2.25) | 2.38 (1.8 to 2.97) | * | 2.28 (.725 to 3.84) |

| | | | | | |
|---|---|---|---|---|---|
| R2 | Other Asian | 48.5 (34.4 to 62.7) | 53.3 (41.6 to 64.9) | * | * |
| D statistic | Other Asian | 1.99 (1.42 to 2.55) | 2.19 (1.67 to 2.7) | * | * |
| R2 | Caribbean | 57.3 (49.4 to 65.2) | 55 (46.6 to 63.4) | 52 (21 to 83) | 64.3 (39.4 to 89.3) |
| D statistic | Caribbean | 2.37 (1.99 to 2.76) | 2.26 (1.88 to 2.65) | 2.13 (.817 to 3.45) | 2.77 (1.31 to 4.23) |
| R2 | Black african | 59.7 (51.2 to 68.2) | 55.1 (47.6 to 62.7) | 3.76 (-14.4 to 21.9) | 29.9 (-6.49 to 66.3) |
| D statistic | Black african | 2.49 (2.05 to 2.93) | 2.27 (1.92 to 2.61) | .37 (-.676 to 1.42) | 1.34 (.169 to 2.51) |
| R2 | Chinese | 63 (40.1 to 85.8) | 67.9 (54.2 to 81.7) | 24.4 (-85.2 to 134) | 67.3 (35.2 to 99.4) |
| D statistic | Chinese | 2.68 (1.39 to 3.98) | 2.98 (2.04 to 3.92) | 1.15 (-4.58 to 6.87) | 2.98 (.787 to 5.18) |
| R2 | Other ethnic group | 48.8 (37.2 to 60.4) | 56.2 (48.4 to 63.9) | 15.4 (-21 to 51.8) | 39.5 (9.11 to 69.8) |
| D statistic | Other ethnic group | 2 (1.54 to 2.47) | 2.32 (1.95 to 2.68) | .849 (-.435 to 2.13) | 1.66 (.605 to 2.71) |
| | | | | | |
| R2 | East Midlands | 59.2 (49.3 to 69) | 64.9 (57.1 to 72.8) | 50.8 (37.4 to 64.1) | 55.6 (38.9 to 72.4) |
| D statistic | East Midlands | 2.46 (1.96 to 2.97) | 2.79 (2.31 to 3.27) | 2.08 (1.52 to 2.63) | 2.29 (1.52 to 3.07) |
| R2 | East of England | 54.7 (46.6 to 62.7) | 52 (44.5 to 59.4) | 47.9 (31.5 to 64.3) | 62 (51.4 to 72.6) |
| D statistic | East of England | 2.25 (1.88 to 2.61) | 2.13 (1.81 to 2.45) | 1.96 (1.32 to 2.61) | 2.61 (2.03 to 3.2) |
| R2 | London | 59.8 (57 to 62.6) | 59.3 (57 to 61.7) | 28.8 (16.4 to 41.2) | 62.9 (55.7 to 70.1) |
| D statistic | London | 2.5 (2.35 to 2.64) | 2.47 (2.35 to 2.59) | 1.3 (.906 to 1.7) | 2.66 (2.25 to 3.07) |
| R2 | North East | 48 (35 to 61.1) | 54.5 (43.5 to 65.6) | 54.2 (19 to 89.4) | 62.9 (23.3 to 102) |
| D statistic | North East | 1.97 (1.45 to 2.48) | 2.24 (1.74 to 2.74) | 2.23 (.647 to 3.81) | 2.66 (.404 to 4.92) |
| R2 | North West | 58.7 (55.2 to 62.2) | 59.5 (56.4 to 62.6) | 53.5 (47.7 to 59.3) | 53.8 (47.8 to 59.9) |
| D statistic | North West | 2.44 (2.27 to 2.61) | 2.48 (2.32 to 2.64) | 2.2 (1.94 to 2.45) | 2.21 (1.94 to 2.48) |
| R2 | South Central | 52.9 (47.7 to 58.1) | 56.6 (52.3 to 60.9) | 41.8 (29.2 to 54.4) | 53.8 (43.3 to 64.3) |
| D statistic | South Central | 2.17 (1.94 to 2.39) | 2.34 (2.13 to 2.54) | 1.73 (1.29 to 2.18) | 2.21 (1.74 to 2.68) |
| R2 | South East | 57.2 (51.8 to 62.6) | 54.4 (49.3 to 59.5) | 44.8 (34.7 to 55) | 60.1 (52.3 to 67.9) |
| D statistic | South East | 2.37 (2.11 to 2.62) | 2.24 (2.01 to 2.47) | 1.84 (1.47 to 2.22) | 2.51 (2.1 to 2.92) |
| R2 | South West | 48 (41.3 to 54.7) | 52.5 (46.6 to 58.3) | 37.2 (25 to 49.4) | 51.9 (42.2 to 61.6) |
| D statistic | South West | 1.96 (1.7 to 2.23) | 2.15 (1.9 to 2.4) | 1.58 (1.16 to 1.99) | 2.13 (1.71 to 2.54) |
| R2 | West Midlands | 54.9 (49 to 60.8) | 54.2 (48.9 to 59.5) | 44.6 (31.8 to 57.5) | 46.8 (34.8 to 58.8) |
| D statistic | West Midlands | 2.26 (1.99 to 2.53) | 2.23 (1.99 to 2.46) | 1.84 (1.36 to 2.32) | 1.92 (1.46 to 2.38) |

| | | | | | |
|---|---|---|---|---|---|
| R2 | Yorkshire & Humber | 62 (53.6 to 70.5) | 68.9 (63.2 to 74.7) | 47.2 (28.8 to 65.7) | 54.4 (39 to 69.9) |
| D statistic | Yorkshire & Humber | 2.62 (2.15 to 3.08) | 3.05 (2.64 to 3.46) | 1.94 (1.22 to 2.65) | 2.24 (1.54 to 2.94) |
| | | | | | |
| R2 | Quintile 1 | 53.7 (49.5 to 57.8) | 53.3 (49.7 to 56.8) | 54.4 (47.7 to 61.1) | 60.7 (55.2 to 66.3) |
| D statistic | Quintile1 | 2.2 (2.02 to 2.39) | 2.19 (2.03 to 2.34) | 2.23 (1.93 to 2.54) | 2.55 (2.25 to 2.84) |
| R2 | 2 | 58.8 (55.2 to 62.4) | 57.5 (53.9 to 61.1) | 41.7 (32.8 to 50.6) | 58.5 (51.7 to 65.2) |
| D statistic | 2 | 2.45 (2.26 to 2.63) | 2.38 (2.21 to 2.55) | 1.73 (1.41 to 2.05) | 2.43 (2.09 to 2.77) |
| R2 | 3 | 56 (52.2 to 59.7) | 56.8 (53.6 to 60) | 46.2 (38.2 to 54.2) | 51.9 (44.9 to 58.9) |
| D statistic | 3 | 2.31 (2.13 to 2.48) | 2.35 (2.19 to 2.5) | 1.9 (1.59 to 2.2) | 2.13 (1.83 to 2.43) |
| R2 | 4 | 53.8 (49.9 to 57.7) | 58.6 (55.6 to 61.6) | 45.2 (37.7 to 52.7) | 54.1 (46.7 to 61.5) |
| D statistic | 4 | 2.21 (2.03 to 2.38) | 2.44 (2.29 to 2.59) | 1.86 (1.58 to 2.14) | 2.22 (1.89 to 2.56) |
| R2 | Quintile 5 | 60.9 (57.9 to 63.9) | 61.7 (59.1 to 64.4) | 37.4 (26.8 to 47.9) | 52.1 (43.5 to 60.7) |
| D statistic | Quintile 5 | 2.55 (2.39 to 2.72) | 2.6 (2.45 to 2.74) | 1.58 (1.23 to 1.94) | 2.13 (1.77 to 2.5) |

**Supplementary Table G. Sensitivity for COVID-19 related death over 97 days in the validation cohort consisting 2,173,056 patients with 1,722 COVID-19 related deaths in the first time period at different risk thresholds of relative risk.**

| top centile | total patients in each centile | age-sex relative risk centile cut off | total deaths in each relative risk centile | cumulative % deaths based on relative risk |
|---|---|---|---|---|
| 1 | 21730 | 15.7 | 390 | 22.65 |
| 2 | 21731 | 10.1 | 188 | 33.57 |
| 3 | 21730 | 7.6 | 125 | 40.82 |
| 4 | 21731 | 6.2 | 94 | 46.28 |
| 5 | 21730 | 5.3 | 80 | 50.93 |
| 6 | 21731 | 4.7 | 74 | 55.23 |
| 7 | 21730 | 4.3 | 41 | 57.61 |
| 8 | 21731 | 4.0 | 44 | 60.16 |
| 9 | 21731 | 3.7 | 41 | 62.54 |
| 10 | 21730 | 3.5 | 33 | 64.46 |
| 11 | 21731 | 3.3 | 31 | 66.26 |
| 12 | 21730 | 3.2 | 34 | 68.23 |
| 13 | 21731 | 3.0 | 27 | 69.80 |
| 14 | 21730 | 2.9 | 21 | 71.02 |
| 15 | 21731 | 2.8 | 22 | 72.30 |
| 16 | 21730 | 2.7 | 16 | 73.23 |
| 17 | 21731 | 2.6 | 22 | 74.51 |
| 18 | 21731 | 2.5 | 15 | 75.38 |
| 19 | 21730 | 2.4 | 22 | 76.66 |
| 20 | 21731 | 2.4 | 15 | 77.53 |
| 21 | 21730 | 2.3 | 9 | 78.05 |
| 22 | 21731 | 2.2 | 23 | 79.38 |
| 23 | 21730 | 2.2 | 13 | 80.14 |
| 24 | 21731 | 2.1 | 14 | 80.95 |
| 25 | 21731 | 2.0 | 11 | 81.59 |

Risk threshold is the centile value giving the cut-off of predicted risk over 97 days for defining each group of relative risk compared with someone of the same age/sex with no risk factors.

Sensitivity is percentage of total deaths over 97 days that occurred within the group of patients above the predicted risk threshold.

**Supplementary Figure A: Graphs of the adjusted hazard ratios for BMI, age and the interaction between age and type 2 diabetes for COVID-19 deaths.**

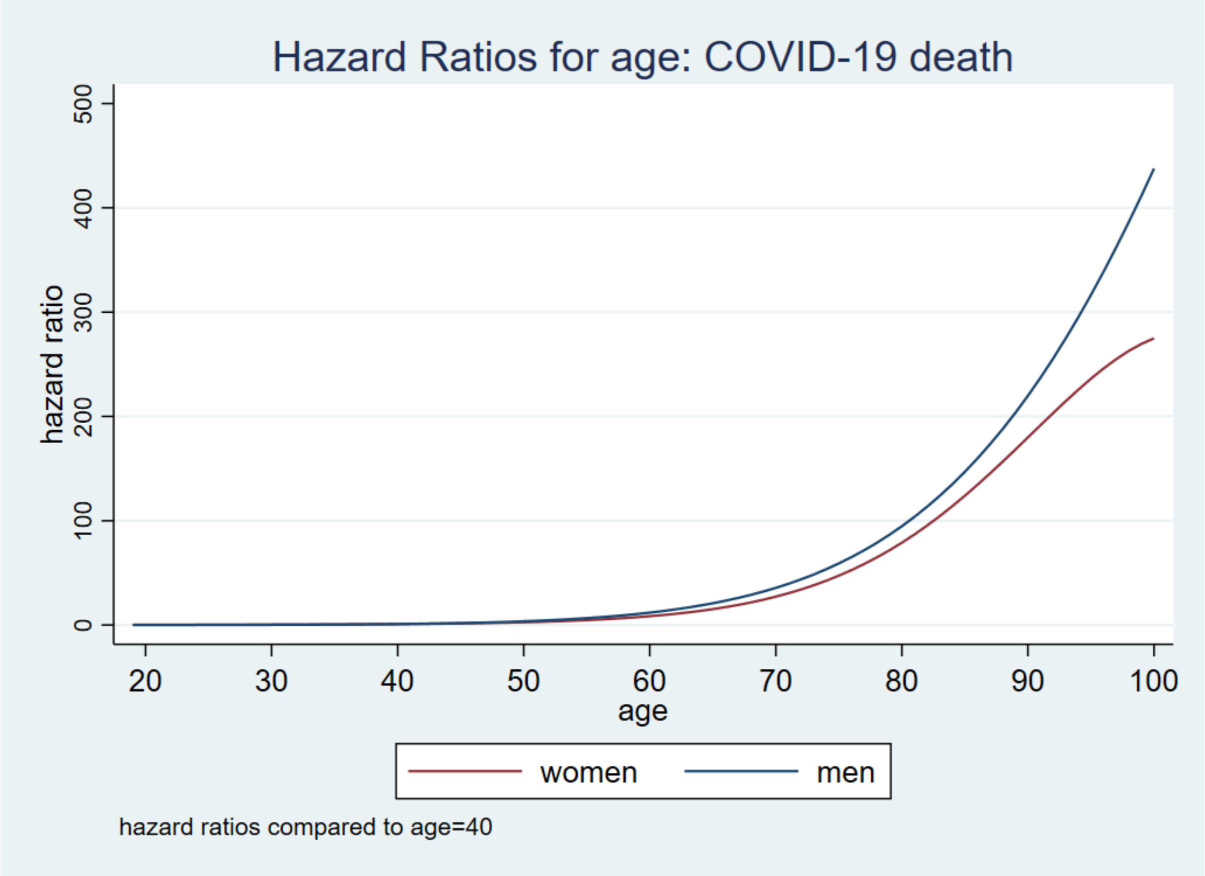


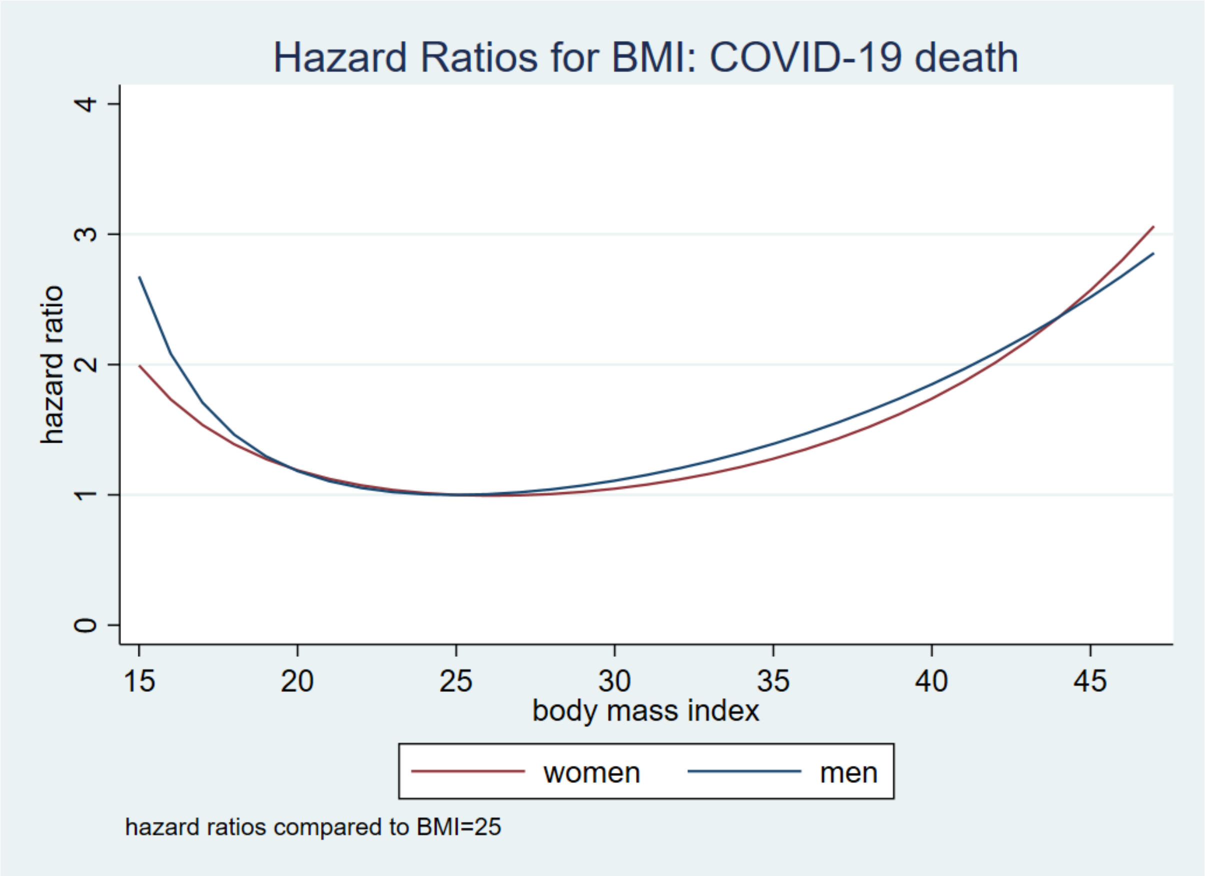

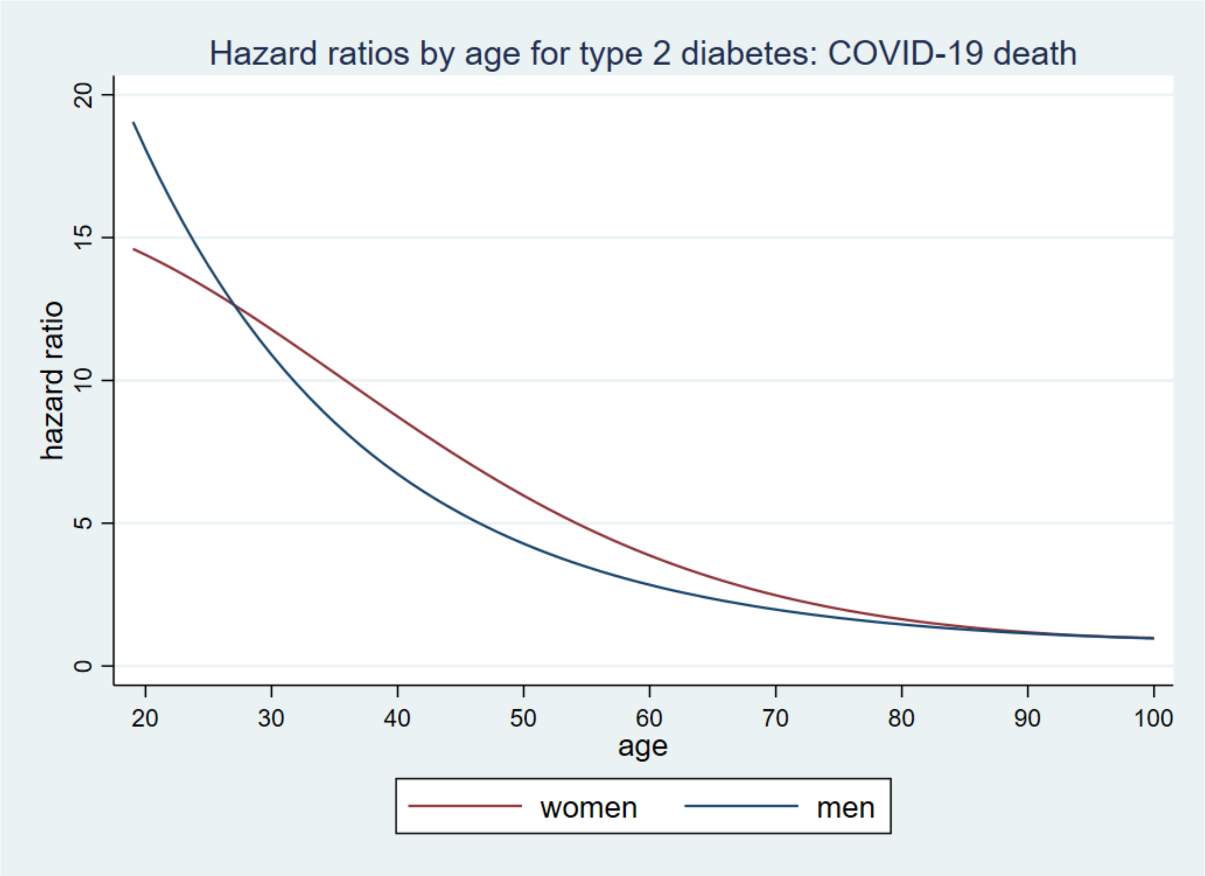
Hazard ratios by age for type 2 diabetes: COVID-19 death
hazard ratio
0
5
10
15
20
20
30
40
50
60
70
80
90
100
age
women
men

**Supplementary Figure B: Graphs of the adjusted hazard ratios for BMI, age and the interaction between age and type 2 diabetes for COVID-19 hospital admissions**

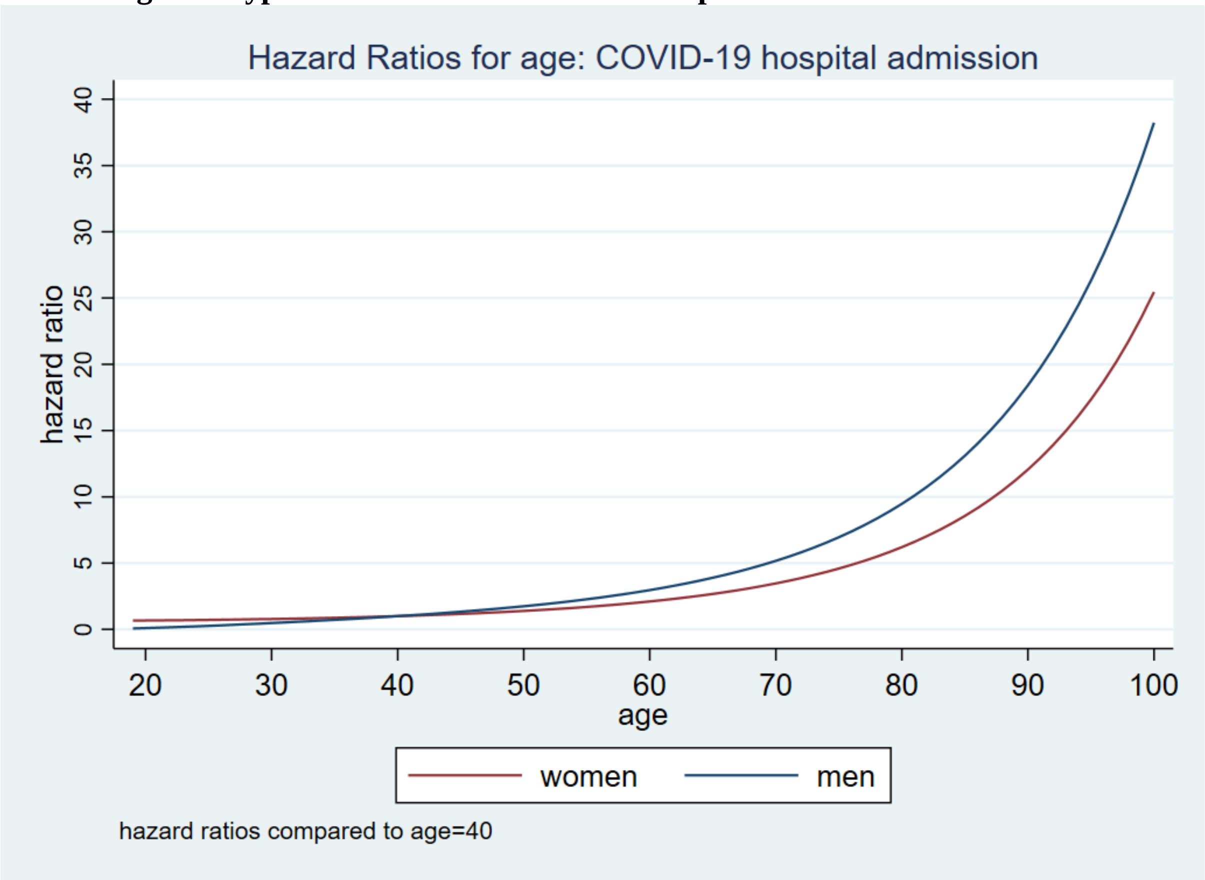


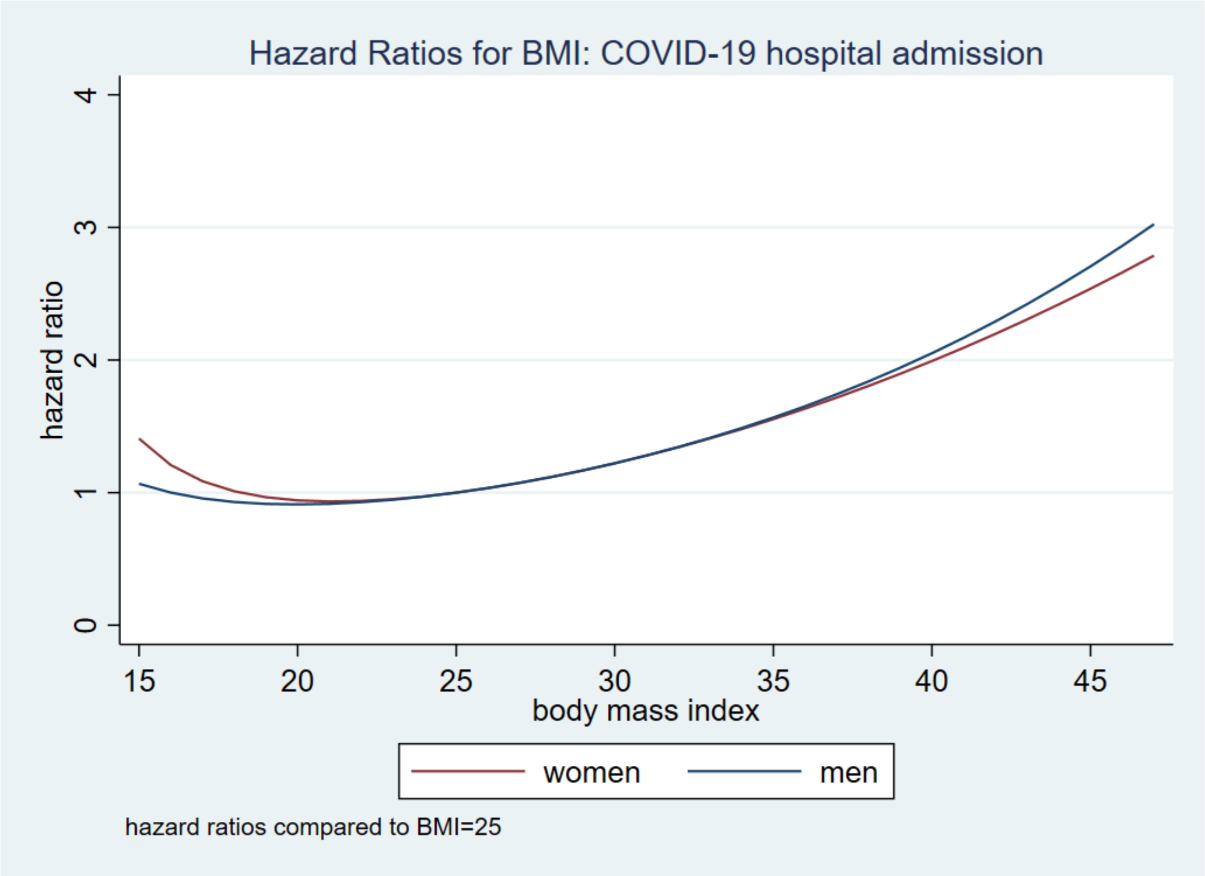

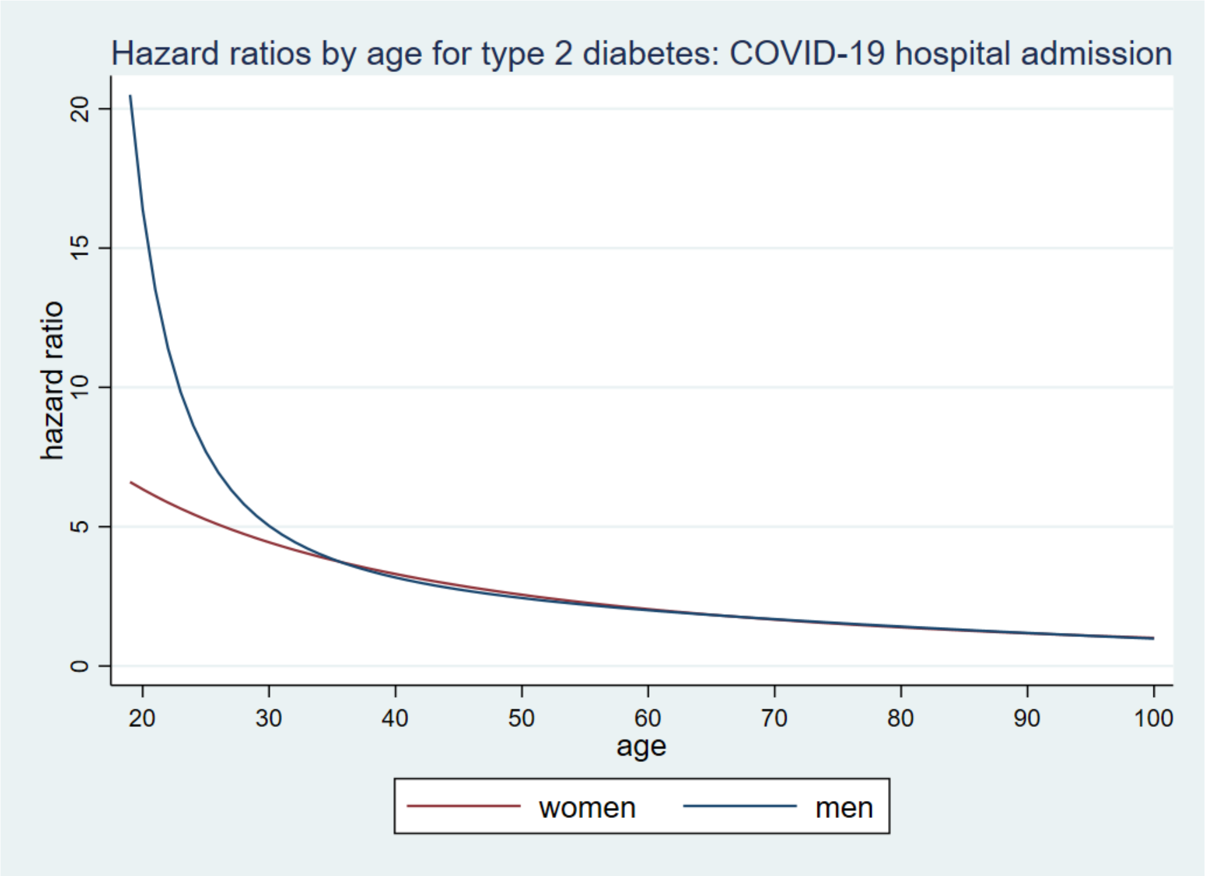
Hazard ratios by age for type 2 diabetes: COVID-19 hospital admission
hazard ratio
0
5
10
15
20
20
30
40
50
60
70
80
90
100
age
women
men

**Supplementary Figure C: Fully adjusted hazard ratios in women for variables for the full model including variables which were not retained in the final model**

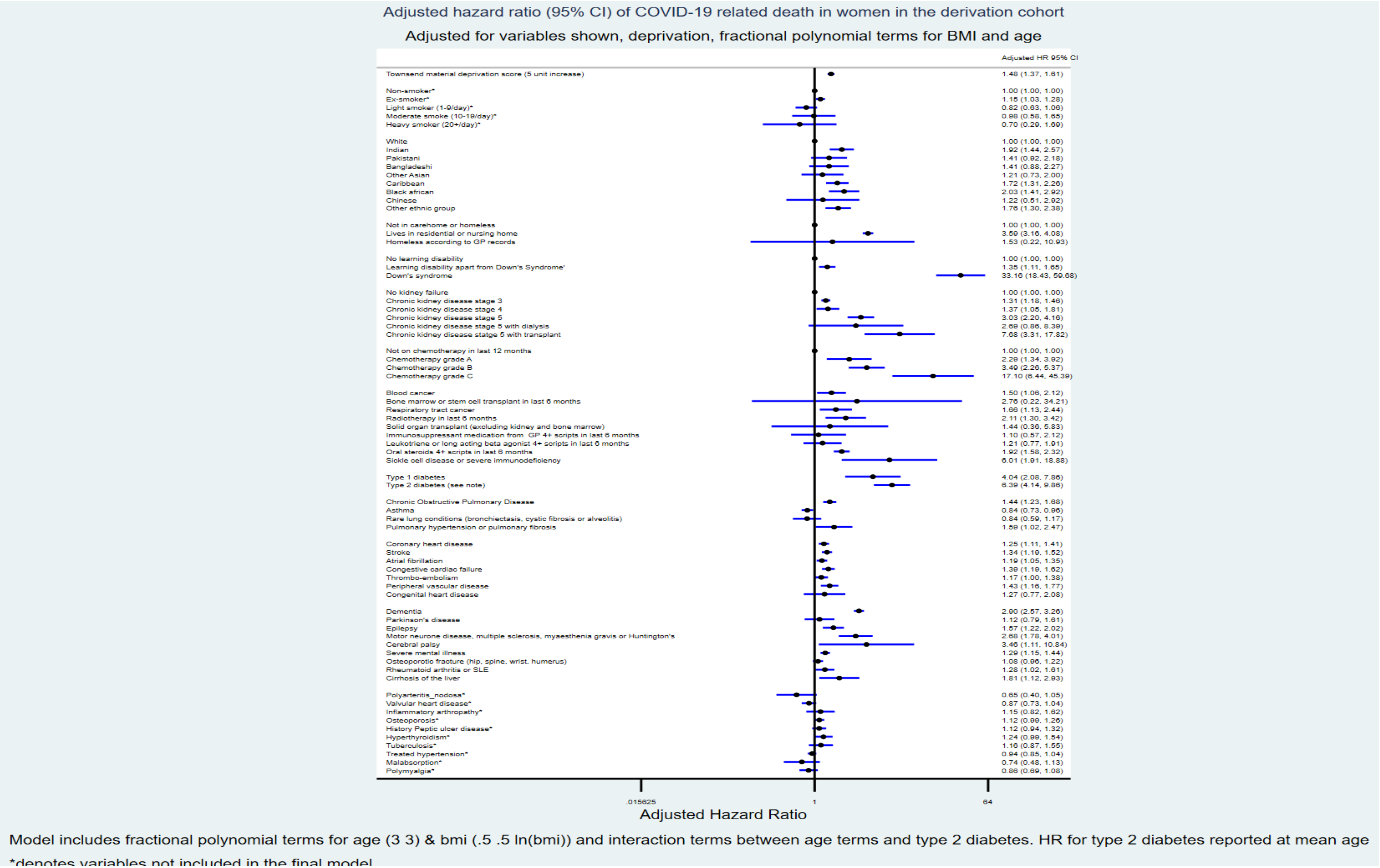

**Supplementary Figure D: Fully adjusted hazard ratios in men for variables for the full model including variables which were not retained in the final model**

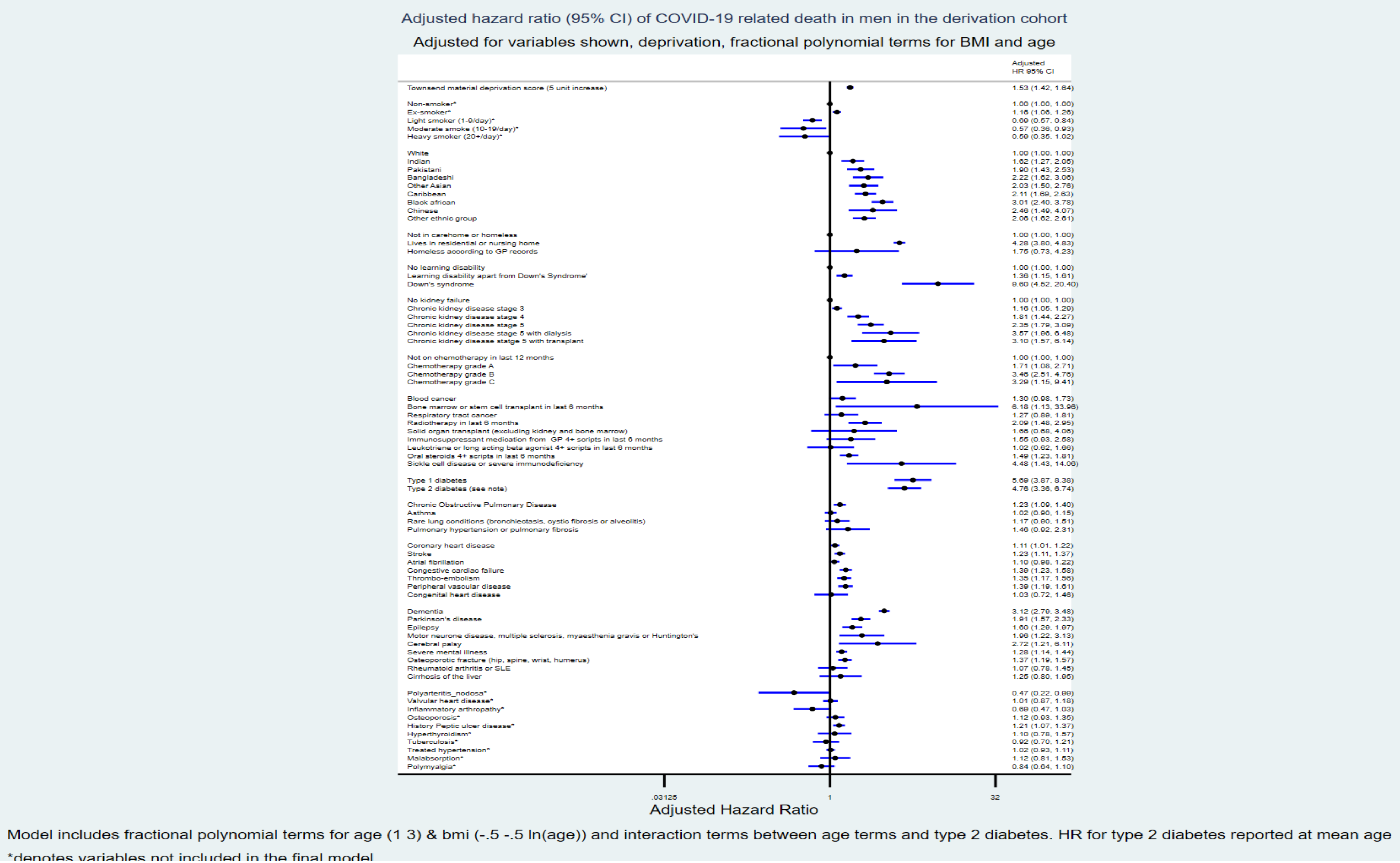

**Supplementary Figure E: Fully adjusted hazard ratios for a combined outcome of either COVID-19 related death or hospital admission in women.**

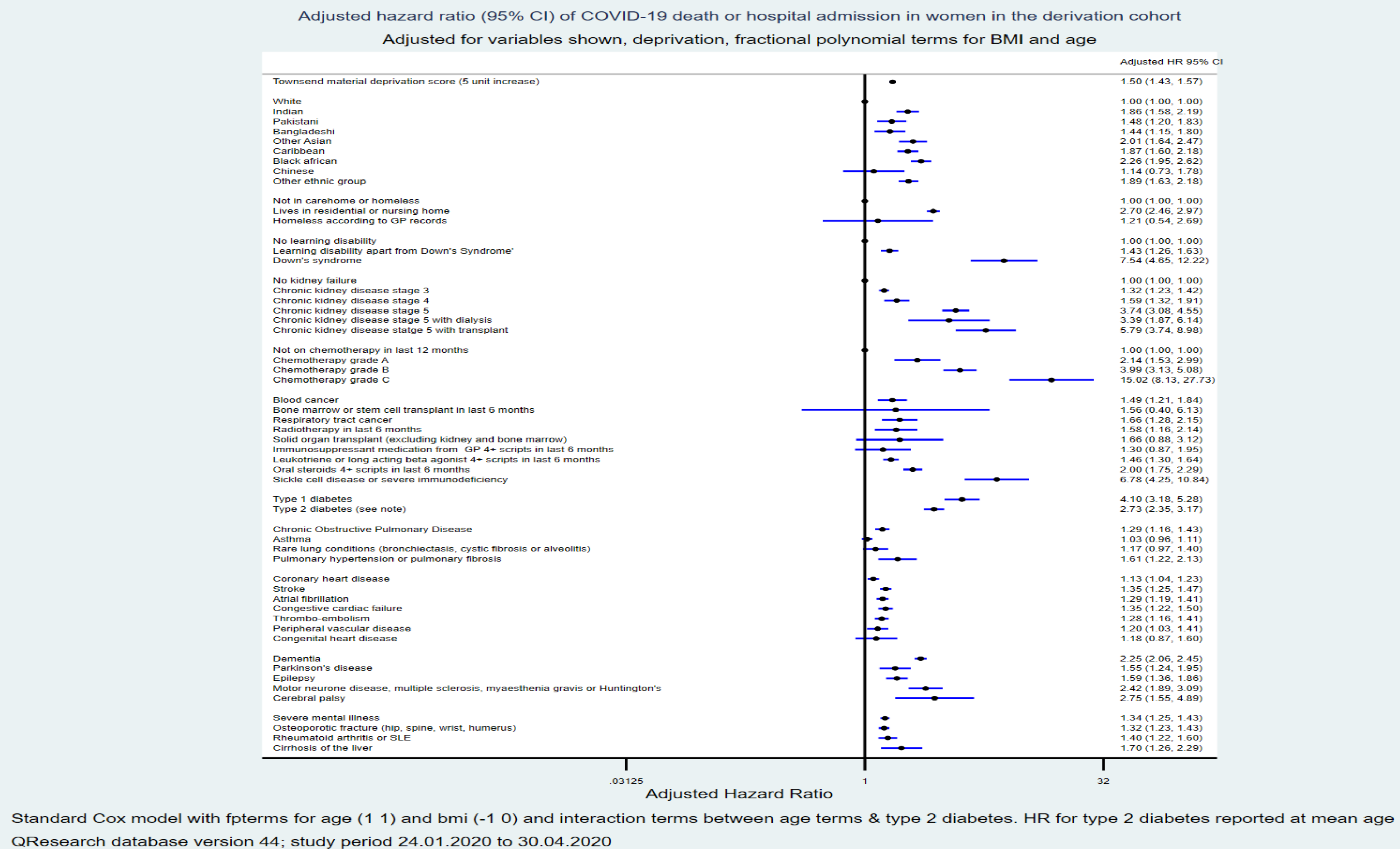

## Supplementary Figure F: Fully adjusted hazard ratios for a combined outcome of COVID-19 related death or hospital admission in men

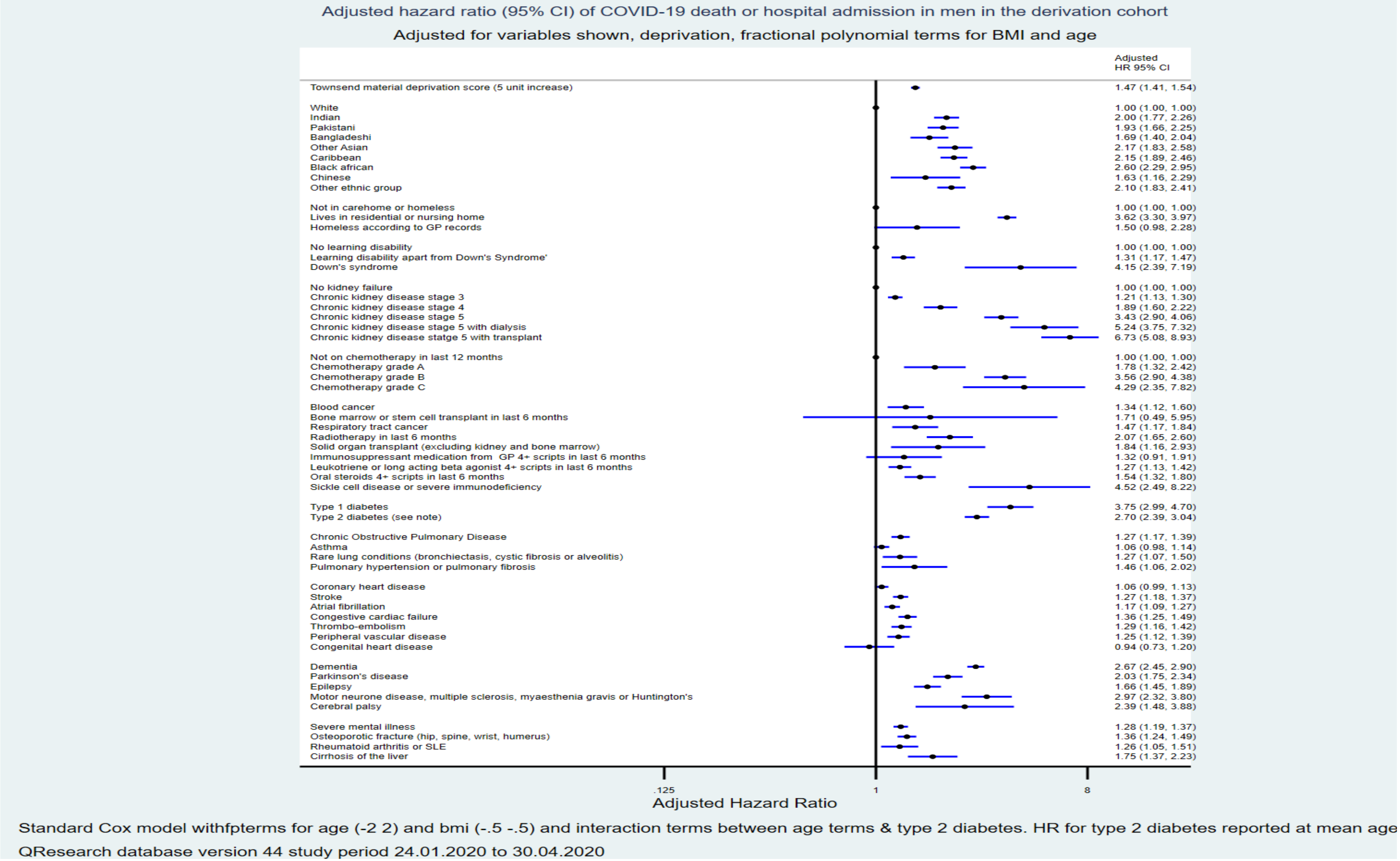

**Supplementary Figure G: Example risk calculation in 55 year old black African man with type 2 diabetes, body mass index of 27.7, and no other risk factors**

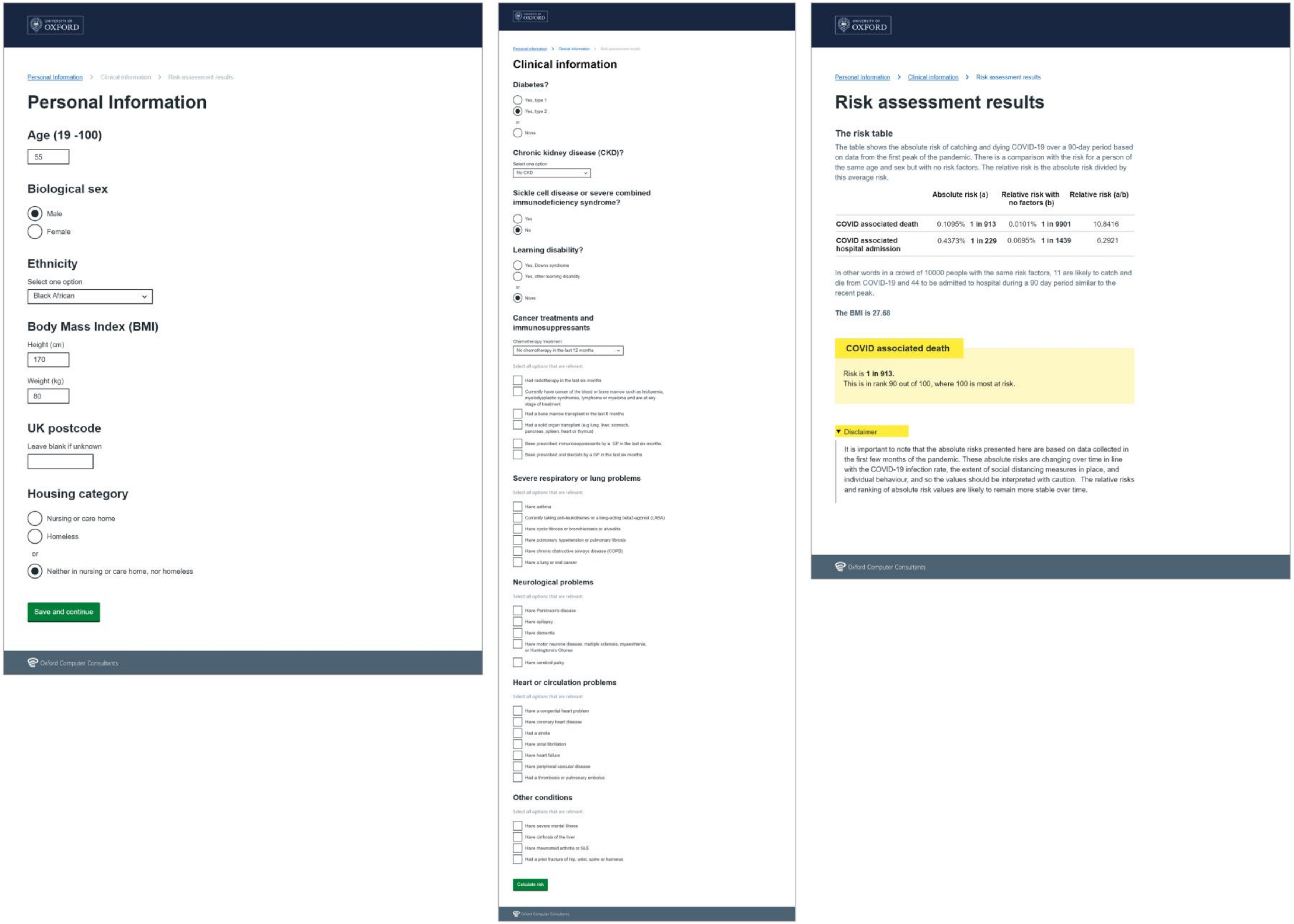

**Supplementary Figure H: Example risk calculation in 30 year old white woman with Down's syndrome and body mass index of 40**

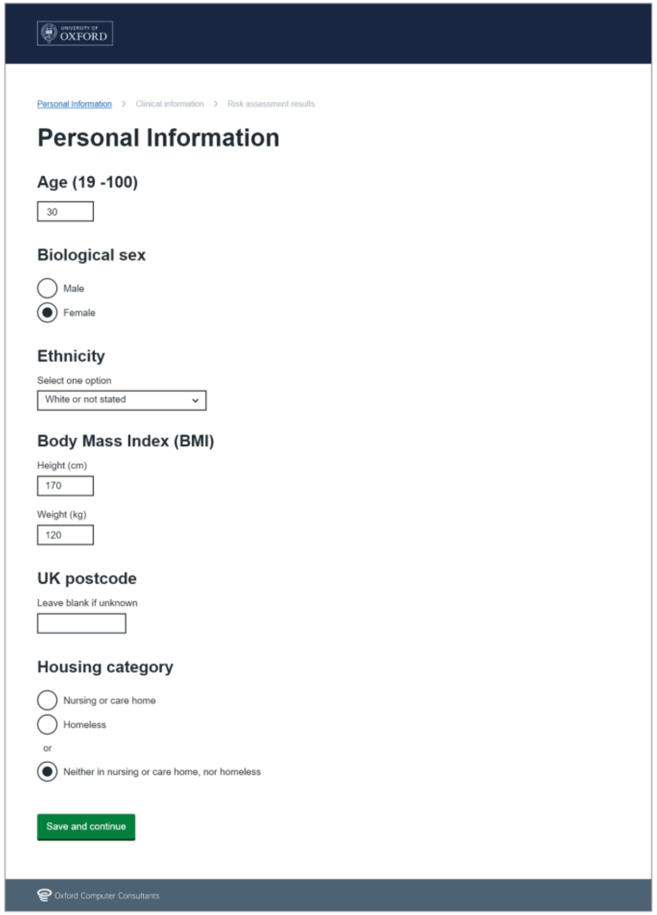

OXFORD

Personal Information > Clinical information > Risk assessment results

## Personal Information

### Age (19 -100)

30

### Biological sex

( ) Male
(•) Female

### Ethnicity

Select one option

White or not stated

### Body Mass Index (BMI)

Height (cm)

170

Weight (kg)

120

### UK postcode

Leave blank if unknown

### Housing category

( ) Nursing or care home
( ) Homeless
or
(•) Neither in nursing or care home, nor homeless

Save and continue

Oxford Computer Consultants

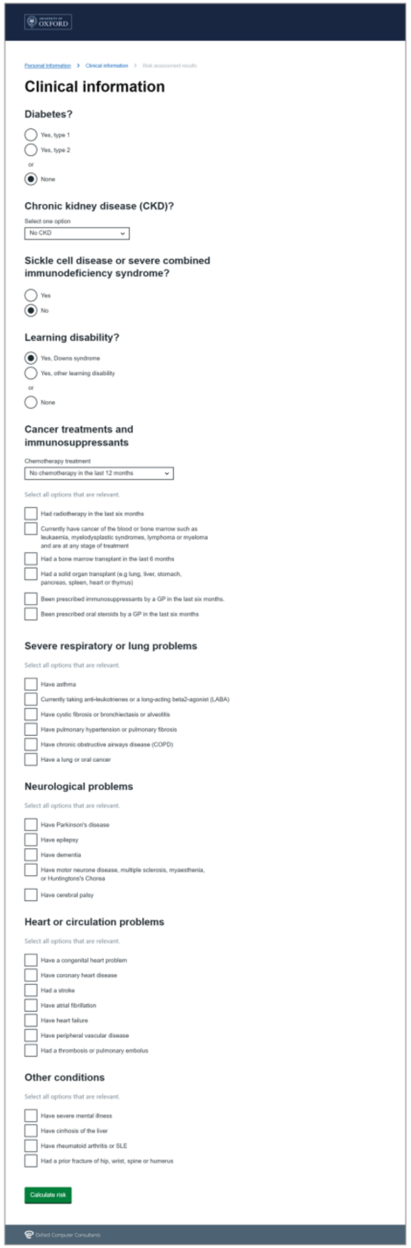

OXFORD

Personal Information > Clinical information > Risk assessment results

## Clinical information

### Diabetes?

( ) Yes, type 1
( ) Yes, type 2
or
(•) None

### Chronic kidney disease (CKD)?

Select one option

No CKD

### Sickle cell disease or severe combined immunodeficiency syndrome?

( ) Yes
(•) No

### Learning disability?

(•) Yes, Downs syndrome
( ) Yes, other learning disability
or
( ) None

### Cancer treatments and immunosuppressants

Chemotherapy treatment

No chemotherapy in the last 12 months

Select all options that are relevant.

- [ ] Had radiotherapy in the last six months
- [ ] Currently have cancer of the blood or bone marrow such as leukaemia, myelodysplastic syndromes, lymphoma or myeloma and are at any stage of treatment
- [ ] Had a bone marrow transplant in the last 6 months
- [ ] Had a solid organ transplant (e.g lung, liver, stomach, pancreas, spleen, heart or thymus)
- [ ] Been prescribed immunosuppressants by a GP in the last six months.
- [ ] Been prescribed oral steroids by a GP in the last six months

### Severe respiratory or lung problems

Select all options that are relevant.

- [ ] Have asthma
- [ ] Currently taking anti-leukotrienes or a long-acting beta2-agonist (LABA)
- [ ] Have cystic fibrosis or bronchiectasis or alveolitis
- [ ] Have pulmonary hypertension or pulmonary fibrosis
- [ ] Have chronic obstructive airways disease (COPD)
- [ ] Have a lung or oral cancer

### Neurological problems

Select all options that are relevant.

- [ ] Have Parkinson's disease
- [ ] Have epilepsy
- [ ] Have dementia
- [ ] Have motor neurone disease, multiple sclerosis, myaesthenia, or Huntingtons's Chorea
- [ ] Have cerebral palsy

### Heart or circulation problems

Select all options that are relevant.

- [ ] Have a congenital heart problem
- [ ] Have coronary heart disease
- [ ] Had a stroke
- [ ] Have atrial fibrillation
- [ ] Have heart failure
- [ ] Have peripheral vascular disease
- [ ] Had a thrombosis or pulmonary embolus

### Other conditions

Select all options that are relevant.

- [ ] Have severe mental illness
- [ ] Have cirrhosis of the liver
- [ ] Have rheumatoid arthritis or SLE
- [ ] Had a prior fracture of hip, wrist, spine or humerus

Calculate risk

Oxford Computer Consultants

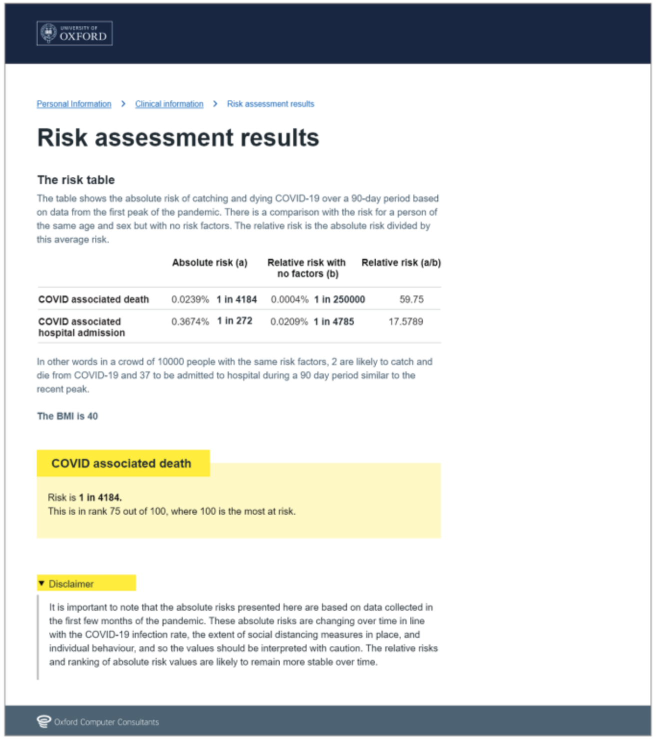

OXFORD

Personal Information > Clinical information > Risk assessment results

## Risk assessment results

### The risk table

The table shows the absolute risk of catching and dying COVID-19 over a 90-day period based on data from the first peak of the pandemic. There is a comparison with the risk for a person of the same age and sex but with no risk factors. The relative risk is the absolute risk divided by this average risk.

| | Absolute risk (a) | | Relative risk with no factors (b) | | Relative risk (a/b) |
|---|---|---|---|---|---|
| **COVID associated death** | 0.0239% | **1 in 4184** | 0.0004% | **1 in 250000** | 59.75 |
| **COVID associated hospital admission** | 0.3674% | **1 in 272** | 0.0209% | **1 in 4785** | 17.5789 |

In other words in a crowd of 10000 people with the same risk factors, 2 are likely to catch and die from COVID-19 and 37 to be admitted to hospital during a 90 day period similar to the recent peak.

**The BMI is 40**

**COVID associated death**

Risk is **1 in 4184.**
This is in rank 75 out of 100, where 100 is the most at risk.

▼ Disclaimer

It is important to note that the absolute risks presented here are based on data collected in the first few months of the pandemic. These absolute risks are changing over time in line with the COVID-19 infection rate, the extent of social distancing measures in place, and individual behaviour, and so the values should be interpreted with caution. The relative risks and ranking of absolute risk values are likely to remain more stable over time.

Oxford Computer Consultants
